# Suppression of Exosomal PD-L1 Induces Systemic Anti-tumor Immunity and Memory

**DOI:** 10.1016/j.cell.2019.02.016

**Published:** 2019-04-04

**Authors:** Mauro Poggio, Tianyi Hu, Chien-Chun Pai, Brandon Chu, Cassandra D. Belair, Anthony Chang, Elizabeth Montabana, Ursula E. Lang, Qi Fu, Lawrence Fong, Robert Blelloch

**Affiliations:** 1 Department of Urology, University of California, San Francisco, San Francisco, CA 94143, USA; 2 Eli and Edith Broad Institute for Regeneration Medicine, University of California, San Francisco, San Francisco, CA 94143, USA; 3 Helen Diller Family Comprehensive Cancer Center, University of California, San Francisco, San Francisco, CA 94143, USA; 4 Department of Medicine, University of California, San Francisco, San Francisco, CA 94143, USA; 5 Division of Molecular Biophysics and Integrated Bioimaging, Lawrence Berkeley National Laboratory, Berkeley, CA 94720, USA; 6 Department of Pathology and Dermatology, University of California, San Francisco, San Francisco, CA 94143, USA; 7 Lead Contact

## Abstract

PD-L1 on the surface of tumor cells binds its receptor PD-1 on effector T cells, thereby suppressing their activity. Antibody blockade of PD-L1 can activate an anti-tumor immune response leading to durable remissions in a subset of cancer patients. Here, we describe an alternative mechanism of PD-L1 activity involving its secretion in tumor-derived exosomes. Removal of exosomal PD-L1 inhibits tumor growth, even in models resistant to anti-PD-L1 antibodies. Exosomal PD-L1 from the tumor suppresses T cell activation in the draining lymph node. Systemically introduced exosomal PD-L1 rescues growth of tumors unable to secrete their own. Exposure to exosomal PD-L1-deficient tumor cells suppresses growth of wild-type tumor cells injected at a distant site, simultaneously or months later. Anti-PD-L1 antibodies work additively, not redundantly, with exosomal PD-L1 blockade to suppress tumor growth. Together, these findings show that exosomal PD-L1 represents an unexplored therapeutic target, which could overcome resistance to current antibody approaches.

## In Brief

Exosomal PD-L1 systemically acts to suppress the anti-tumor immune response, and its genetic blockage promotes T cell activity in the draining lymph node to induce systemic anti-tumor immunity and memory.

## Graphical Abstract

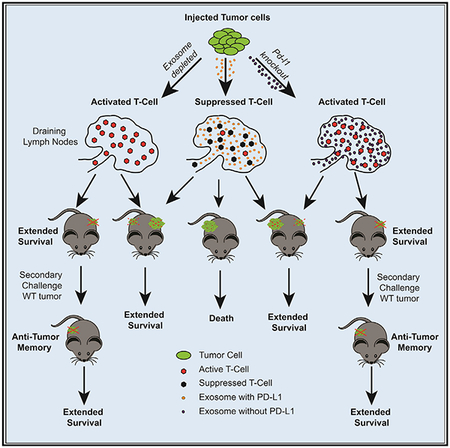

## INTRODUCTION

Immunotherapy has revolutionized cancer therapy (Chen and Mellman, 2017). Immune checkpoint protein inhibitors, such antibodies against PD-L1 (aka CD274) and PD-1 (aka PDCD1), have shown effectiveness against a large number of cancer types, including melanoma, non-small-cell lung cancer, and renal cancer. This response includes durable remissions many patients who had previously failed multiple other therapeutic strategies. However, even in these cancers, only 10%–30% patients respond to anti-PD-L1/PD-1 therapy (Page et al., 2014). In other cancers, such as prostate cancer, responses are rare (Goswami et al., 2016; Sharma et al., 2017). The basis differential therapeutic success between patients and between cancers remains largely unknown.

PD-L1 is a membrane bound ligand found on the cell surface of many cell types that is upregulated in the setting of inflammation and/or a number of oncogenic lesions (Topalian et al., 2015). It binds the PD-1 receptor on immune T cells, leading to Sh2p-driven dephosphorylation of the T cell receptor and its co-receptor CD28, thereby suppressing antigen-driven activation of T cells (Hui et al., 2017; Yokosuka et al., 2012). This mechanism normally keeps inflammatory responses in check, and *Pd-l1* knockout mice develop autoimmune-like diseases (Francisco et al., 2010). However, tumor cells can co-opt this mechanism to evade immune destruction. Therapeutic antibodies to PD-L1 and PD-1 block this interaction, which can then reactivate the anti-tumor immune response (Chen and Mellman, 2017).

It is generally thought that PD-L1 functions within the tumor bed, where cell-surface PD-L1 is directly interacting with PD-1 on the surface of tumor-infiltrating lymphocytes (TILs) (Mellman et al., 2011). However, PD-L1 also can be found on surface of extracellular vesicles (EVs). Furthermore, EV PD-L1 levels have been associated with tumor progression (Chen et al., 2018; Ricklefs et al., 2018; Theodoraki et al., 2018; Yang et al., 2018). Whether extracellular PD-L1 can promote tumor progression by inducing a local and/or systemic immunosuppression is unknown.

EVs are heterogeneous (Tkach et al., 2018). A particular form of EVs is exosomes, which derive from the endocytic pathway (van Niel et al., 2018). As endosomes mature, vesicles bud inward and are released in the lumen forming intravesicular bodies within the late endosomes. These late endosomes are also called multivesicular bodies (MVB). MVBs can either fuse with lysosomes for degradation and recycling of contents or fuse with the plasma membrane releasing the intravesicular bodies extra-cellularly, which are then called exosomes. Exosomes can be differentiated from other EVs based on their size, morphology, density, marker expression, and dependency for specific enzymes for their biogenesis. Key enzymes in their biogenesis include NSMASE2 (aka SMPD3), which promotes budding of intravesicular vesicles, and RAB27A, which is involved in the fusion of the MVB to the plasma membrane (Kosaka et al., 2010; Ostrowski et al., 2010). Genetic manipulation of these enzymes provides an opportunity to dissect the role of exosomes *in vivo*.

While evaluating mechanisms regulating levels of PD-L1 in different tumors, we discovered that cancer cells can secrete a vast majority of their PD-L1 on exosomes rather than present PD-L1 on their cell surface. Using genetic knockouts for *Rab27a* and *nSMase2* and exogenously introduced exosomes, we show that exosomal PD-L1 from tumor cells promote tumor growth in an immune-dependent fashion. Exosomal PD-L1 suppresses T cell function *in vitro* and *in vivo* at the site of the draining lymph node. Exosomal PD-L1 appears to be resistant to anti-PD-L1 as a prostate cancer syngeneic model that is unresponsive to such therapy, is dependent on both PD-L1 and exosomes for their growth. Remarkably, even the transient presence of cancer cells deficient in exosomal PD-L1 results in long-term, systemic immunity against the cancer. A role for exosomal PD-L1 is also seen in a syngeneic colorectal model. In this model, anti-PD-L1 acts additively, not redundantly, with the suppression of PD-L1 secretion. These findings have significant implications for immunotherapeutic approaches to cancer therapy.

## RESULTS

### Differential Secretion of PD-L1 between CancerCell Lines

**Figure 1.**
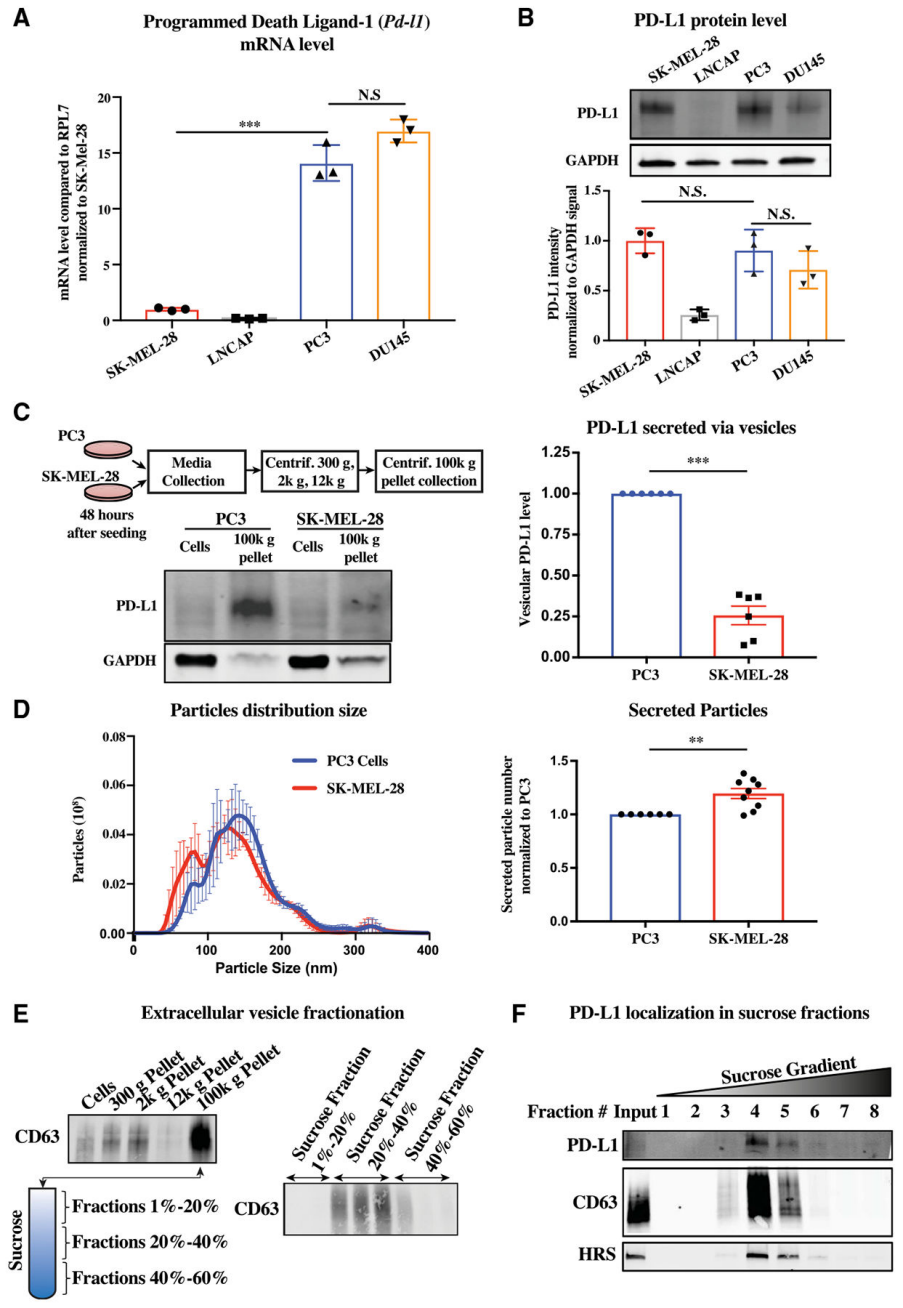
Differential Secretion of PD-L1 in PC3 versus SK-MEL-28 Cells (A) qRT-PCR of *Pd-l1* mRNA levels in one melanoma cell line (SK-MEL-28) and three prostate cancer cell lines (LNCAP, PC3, and DU145). n = 3. (B) Western analysis of cellular levels of PD-L1 in the same cells as (A). Top: Representative western blot. Bottom: Quantification of three independent western blots. (C) Top left: Schematic for crude isolation of secreted vesicles by sequential centrifugation of media through increasing centrifugal forces and finally pelleted at 100k g. Bottom left: Representative western blot of PD-L1 in cells versus secreted vesicles of either PC3 or SK-MEL-28 cells. Right: Quantification. n = 6. (D) Nanoparticle tracking of size and quantity of secreted vesicles produced by PC3 and SK-MEL-28 cells. Left: Density plot for size. Right: Total vesicle number based on integration under the curve. n = 9. (E) Schematic of steps involved in exosome purification including sequential centrifugations of supernatant at increasing gravitation force and then 100k g pellet run on sucrose gradient. Location of exosomes is followed by a western blot for CD63. (F) Western blot for PD-L1 and exosome markers CD63 and HRS in different sucrose gradient fractions. n = 3. (A and B) Error bars represent SD. (C and D) Error bars represent SEM. *p < 0.05; **p < 0.01; ***p < 0.001 (Student’s t test). See also Figures S1 and S2.

It has been reported that surface PD-L1 levels are low in prostate cancer cell lines and primary prostate tumor tissue, potentially explaining the general lack of therapeutic response to anti-PDL1 blockade (Martin et al., 2015; Zang et al., 2007). We sought to determine whether a transcriptional, post-transcriptional, translational, or post-translational mechanism underlies the reduced levels by comparing prostate cancer cell lines (PC3, DU145, LNCaP) to a melanoma cell line (SK-MEL-28). Measurements of mRNA and protein levels showed discordance between mRNA and protein levels across the different cancer cell lines. qRT-PCR showed a 15-fold increase of *Pd-l1* mRNA levels in PC3 and DU145 relative to SK-MEL-28; LNCaP showed a near absence of transcripts (Figure 1A). In contrast to mRNA levels, western analysis showed similar cellular PD-L1 protein levels in PC3 and DU145 cells as SK-MEL-28 (Figure 1B); protein was undetectable in LNCaP cells. We asked whether the discordance in mRNA and protein levels between PC3 and SK-MEL-28 could be explained by differences in protein translation. Translation rates were determined by polysome profiling, a method by which transcripts bound by many ribosomes reflective of a high translation rate are separated from transcripts bound by one or a few ribosomes reflective of a low translation rate (Chassé et al., 2017). The two populations were separated on a sucrose gradient, and then the bound mRNA was measured by qRT-PCR (Figure S1A). This analysis showed an equal distribution of the *Pd-l1* RNA across the fractions in PC3 and SK-MEL-28 (Figure S1B). Thus, differences in translation rates cannot explain the discordance between mRNA and protein levels between the lines.

Next, we evaluated potential differences in protein degradation. The two main pathways for protein degradation are the lysosome and the proteasome (Ciechanover, 2005). The small molecule Bafilomycin A1 (BafA1) inhibits lysosomal activity by blocking the V-ATPase hydrogen pump and thus acidification of the lysosome (Mauvezin and Neufeld, 2015). An increase in the protein LC3B, a known target of the lysosome, confirmed effectiveness of the small molecule on PC3 and SK-MEL-28 cells. However, levels of PD-L1 in both lines were unaltered, implying little turnover of PD-L1 by the lyso-some in these cells (Figures S1C and S1D). The small molecule MG132 suppresses proteasome activity by blocking the 26S proteasome complex and, consequentially, proteolysis (Lee and Goldberg, 1998). The proteasome recognizes ubiquitin side chains on its targets (Lee and Goldberg, 1998). An increase in the presence of ubiquitinated proteins confirmed the effectiveness of MG132 on the two cells lines. Again though, PD-L1 protein levels were minimally affected, and, if anything, the difference of the two lines was opposite from expected as PD-L1 levels were slightly up in SK-MEL-28, but not in PC3, cells (Figures S2E and S2F). These results show that differences in protein stability do not explain the discordance in mRNA and protein levels.

Next, we considered the possibility that PD-L1 may be differentially secreted from cells in the form of membrane vesicles. Extra-cellular vesicles can be enriched using sequential centrifugation transferring supernatant through increasing gravitational forces to remove cellular debris and apoptotic bodies, before finally pelleting at 100,000 gravitational forces (100k g) (Xu et al., 2016). Western analysis showed 2- to 3-fold more PD-L1 in the 100k g fraction of PC3 cells relative to SK-MEL-28 cells (Figure 1C). This difference could have been due to more PD-L1 being loaded per vesicle or release of more vesicles. A nanoparticle tracking instrument can track the size and number of vesicles by using light diffraction off particles moving under Brownian motion (Carr and Warren, 2012). Analysis of conditioned media from the two cell types showed a slightly elevated total vesicle count in SK-MEL-28 (Figure 1D). Thus, even though PC3 and SKMEL-28 cells had similar levels of cellular PD-L1 protein, PC3 cells packaged greater amounts of PD-L1 into extracellular vesicles. This difference appears to underlie the discordance between mRNA and protein levels between the two cell lines.

*In vivo*, tumor cells can mediate adaptive resistance by upregulating PD-L1 in response to interferon-gamma (IFNγ) released by the cytotoxic T lymphocytes within the tumor bed (Garcia-Diaz et al., 2017). Therefore, we asked whether, in addition to up-regulating cellular PD-L1, IFNγ may increase the secretion of PD-L1. Treatment of cancer cells with IFNγ led to an increase in PD-L1 in both the cellular and 100k g fractions (Figure S2A). The increase was proportionally similar between the two fractions, suggesting no direct impact of IFNγ on PD-L1 secretion (Figure S2B). In addition, IFNγ did not increase the number of vesicles secreted (Figure S2C). Thus, similar to the cell-surface membrane PD-L1, extracellular vesicular PD-L1 increases in response to IFNγ.

### PD-L1 Is Specifically Secreted within Exosomes

Extracellular vesicles come in multiple forms differing in size, density, protein markers, and biogenesis (Xu et al., 2016). Given that PD-L1 is endocytosed from the surface of cells (Burr et al., 2017; Mezzadra et al., 2017), we hypothesized that PD-L1 is being specifically secreted in the form of exosomes. Exosomes can be enriched relative to other vesicles based on their density by spinning the crude 100k g pellet on a sucrose gradient (Shurtleff et al., 2016). The exosomal marker CD63 traveled in the 20%–40% sucrose fractions (Figure 1E). PDL1 and the additional exosomal marker HRS colocalized with CD63 (Figure 1F). These data support that PD-L1 is packaged in exosomes.

**Figure 2.**
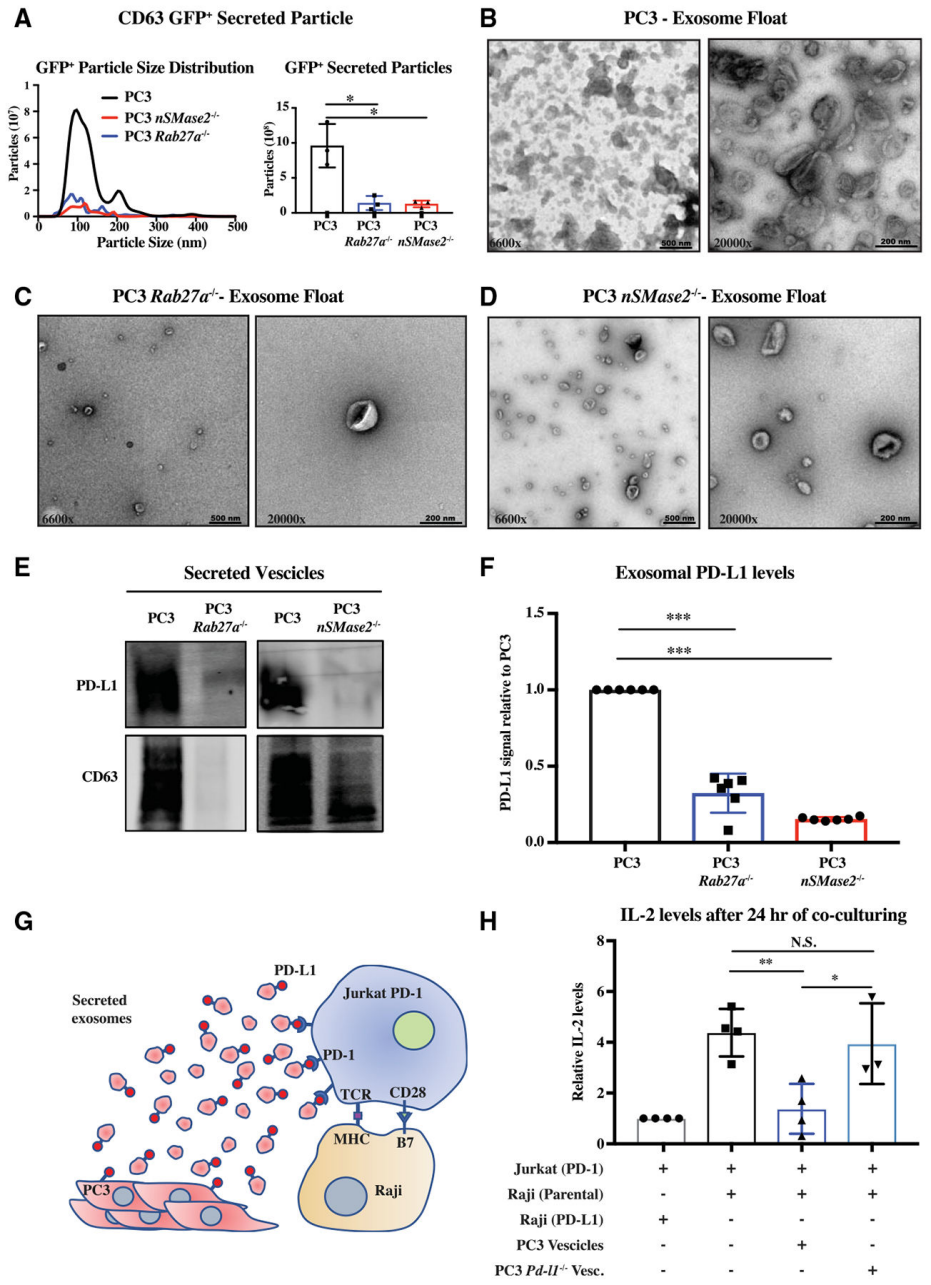
PD-L1 Is Secreted in the Form of *Rab27a*- and *nSMase2*-Dependent Exosomes (A) Nanoparticle tracking of size and quantity of GFP^+^ vesicles from WT and *Rab27a* null PC3 cells expressing CD63-GFP. Left: Density trace. Right: Integration under curve for total particles. n = 3. (B–D) Electron microscopy images of WT (B), *Rab27a* null (C), and *nSMase2* (D) null PC3 exosomes, respectively, purified on sucrose gradient. (E) Representative western for PD-L1 and CD63 in WT PC3, *Rab27a* null, and *nSMase2* null PC3 cells. (F) Quantification of PD-L1 in WT PC3, *Rab27a* null, and *nSMase2* null PC3 cells. n = 6. (G) Schematic of *in vitro* functional assay of exosomal PDL1. (H) Quantification of IL-2 released by Jurkat cells following Raji presentation of super-antigen. Jurkat (PD1), Jurkat T cells overexpressing PD1; Raji (Parental), Raji B cells expressing low levels of PD-L1; Raji (PD-L1), Raji B cells overexpressing PD-L1; PC3 vesicles, 100k g pellet from conditioned media of WT PC3 cells; PC3 *Pd-l1*^−*/*−^ Vesc, 100k g pellet from conditioned media of *Pd-l1* null PC3 cells. n = 4. (A, F, and H) Error bars represent SD. *p < 0.05; **p < 0.01; ***p < 0.001 (Student’s t test). See also Figures S3 and S4.

Next, to determine if PD-L1 is specifically found in exosomes, we took a genetic approach to remove exosomes. We focused on the *Rab27a* and *nSMase2* genes using CRISPR/Cas9-mediated mutagenesis to knock them out in PC3 cells (Figures S3A–S3C). Deletion of these two genes did not affect the proliferation of PC3 cells (Figures S3D and S3E). To follow exosomes, we ectopically expressed CD63-GFP in the three genetic backgrounds. Measurement of the conditioned media showed an almost complete absence of CD63-GFP+ particles arising from the two mutant lines versus wild-type (WT) (Figure 2A). To further evaluate the effect of the *Rab27a* and *nSMase2* deletion on exosomes, we performed electron microscopy on sucrose gradient concentrated particles. The resultant images showed very few exo-some-like particles in the *Rab27a* and *nSMase2* null samples, with a greater loss in the *Rab27a* null cells (Figures 2B–2dD). Consistent with these images, western analysis showed an absence of endogenous CD63 in the 100k g preps from *Rab27a* null cells and a small amount remaining in the *nSMase2* null cells (Figure 2E). In contrast to CD63 levels, *nSMase2* showed a complete absence, while *Rab27a* null cells showed a dramatic reduction in PD-L1 in the 100k g fraction (Figure 2E). This difference appears to be due to an additional role for NSMASE2 on *Pd-l1* transcription (Figures S3F and S3G). Together, these data show critical roles for both *Rab27a* and *nSMase2* in exosome biogenesis and PD-L1 secretion.

Given that PD-L1 is a *trans*-membrane protein, exosomal PDL1 must arise from the limiting membrane of the late endosome. The limiting membrane of the late endosome initially arises from the endocytosis of the plasma membrane. However, material provided directly from the ER and Golgi as well as cytoplasm is added as the resultant endosome matures (van Niel et al., 2018). Flow cytometry and immunofluorescence showed the presence of PD-L1 on both the surface and within vesicle-like structures inside PC3 cells (Figures S4A and S4B). Using standard cell fractionation techniques, PD-L1 and CD63 co-localized in the sucrose light, or endolysosomal fraction (Shurtleff et al., 2016) (Figure S4C). To determine whether exosomal PD-L1 directly arises from the ER or early Golgi, we took advantage of the fact that PD-L1 is glycosylated (Li et al., 2016). This glycosylation was easily removed with the amidase PNGaseF, consistent with it being a N-linked oligosaccharide chain (Figure S4D). In contrast, the majority of cellular and all the exosomal PD-L1 was resistant to EndoH cleavage, consistent with maturation of the oligosaccharide chain in the distal Golgi (Stanley, 2011). Therefore, exosomal PD-L1 does not appear to come directly from the ER or early Golgi. To measure the plasma membrane as a source, we performed a cell-surface biotinylation assay (Figure S4E). Biotin-labeled PD-L1 was found in the cells and exosomes of treated cells, showing that exosomal PD-L1 originates from the surface of the PC3 cells.

### Exosomal PD-L1 Suppresses T Cell Activation *In Vitro*

Next, we asked whether exosomal PD-L1 could function similarly to cell-surface PD-L1 in the suppression of T cell activation. PD-L1 function can be measured *in vitro* in the setting of Raji B cell presentation of antigen to Jurkat T cells (Hui et al., 2017). Normally, this presentation would activate T cells, which can be measured by secretion of interleukin-2 (IL-2). However, if PD-L1 is exogenously expressed in the Raji B cells and PD-1 is exogenously expressed in the Jurkat T cells, T cell activation is suppressed (Hui et al., 2017). In this setting, we asked whether exosomal PD-L1 from PC3 cells could replace exogenous expression of PD-L1 on the Raji B cells (Figure 2G). As expected, in the absence of exogenous expression of PD-L1 in Raji B cells, IL-2 secretion was high (Figure 2H, compare first two bars). However, the introduction of the PC3 100k g extracellular fraction re-repressed IL-2 secretion showing that PC3 vesicles can replace Raji cell PD-L1 expression (Figure 2H, third bar). To determine whether exosomal PD-L1 was responsible for this effect, we used CRISPR/Cas9 editing to delete the *Pd-l1* gene in the PC3 cells (Figure S4F). Deletion of the *Pd-l1* gene did not affect the proliferation of the PC3 cells or alter the number of vesicles released (Figures S4G and S4H). However, introduction of the *Pd-l1* null PC3 100k g extracellular fraction failed to repress IL2 secretion (Figure 2H, fourth bar). These data show that exosomal PD-L1 does function to suppress T cell activation *in vitro*.

### Exosomal PD-L1 Promotes Tumor Progression

**Figure 3.**
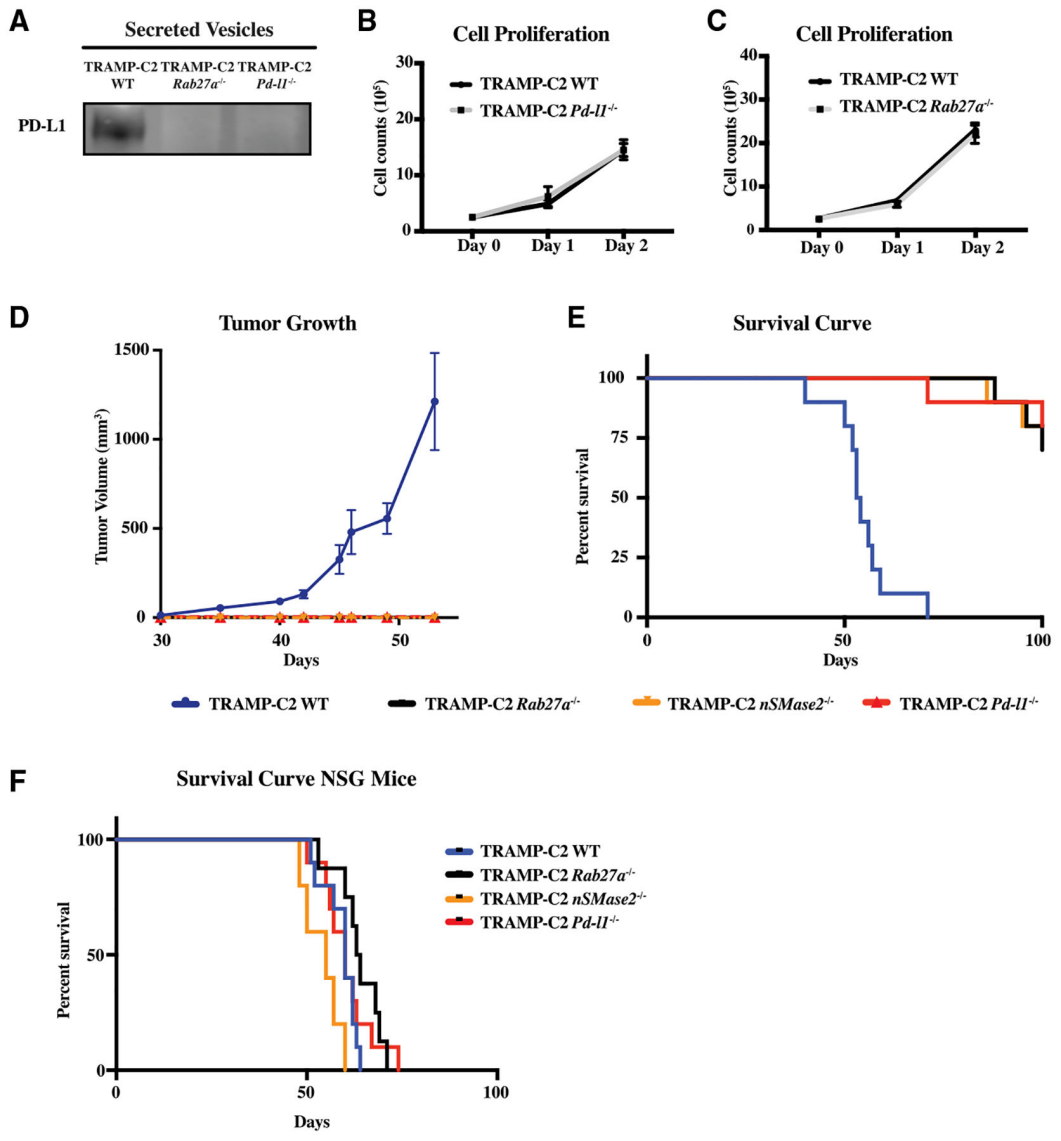
Exosomal PD-L1 Suppresses Tumor Progression in Syngeneic Prostate Cancer Model (A) Western blot for PD-L1 in WT, *Rab27a* null, and *Pd-l1* null TRAMP-C2 100k g extracellular fraction. (B) Cell counts over time for *Pd-l1* null versus WT TRAMP-C2 cells. n = 3. Error bars represent SD. (C) Cell counts over time for *Rab27a* null versus WT TRAMP-C2 cells. n = 3. Error bars represent SD. (D) Tumor growth volume over time following subcutaneous injection of 1 × 10^6^ WT, *Rab27a* null, or *Pd-l1* null TRAMP-C2 cells into immuno-competent B6 mice. n = 5 for each genotype. Error bars represent SEM. Tramp WT versus TRAMP-C2 *Rab27a* null, p < 0.001. Tramp WT versus TRAMP-C2 *Pd-l1* null, p < 0.0001. Tramp WT versus TRAMP-C2 *nSMase2* null, p < 0.001 (twoway ANOVA test). (E) Mouse survival curve following injection of cells like in (D). n = 10 for each genotype. Tramp WT versus TRAMP-C2 *Rab27a* null, p < 0.001. Tramp WT versus TRAMP-C2 *Pd-l1* null, p < 0.001. Tramp WT versus TRAMP-C2 *nSMase2* null, p < 0.001 (log rank test). (F) Mouse survival curve following NOD-SCID IL2rg^−/−^ (NSG) mice injected with 1 × 10^6^ WT, *Rab27a* null, and *Pd-l1* null TRAMP-C2 cells. n = 10 for *Pd-l1* null and WT genotypes, n = 8 for *Rab27a* null genotype, n = 5 for *nSMase2* null genotype. See also Figure S5.

Given its ability to suppress T cell activation *in vitro*, we wanted to know whether exosomal PD-L1 can function *in vivo* to promote tumor progression. To do so, we turned to a syngeneic model of prostate cancer, the TRAMP-C2 model (Foster et al., 1997). This preclinical model, like human prostate cancer, is resistant to anti-PD-L1 blockade (Yu et al., 2012). CRISPR/Cas9-mediated deletion of *Rab27a* (Figure S5A) and *Pd-l1* (Figure S5B) resulted in a loss of PD-L1 in the EV fraction (Figure 3A). Loss of Rab27a did not influence cell-surface PD-L1 levels, nor did loss of *Pd-l1* influence exosome production (Figures S5C and S5D). Like with the PC3 cells, deletion of *Rab27a* or *Pd-l1* did not affect the proliferation of the cells (Figures 3B and 3C). The WT, *Rab27a* null, and *Pd-l1* null TRAMP-C2 cells were injected into the flanks of C57BL6/J syngeneic mice and were followed for over 4 months. All mice injected with the WT TRAMP-C2 cells had visible tumors by around 35 days and had to be euthanized between 40 and 71 days (Figures 3D and 3E). In contrast, all mice injected with *Rab27a* null or *Pd-l1* null TRAMP-C2 cells showed no tumor growth during the same time period (Figure 3D). Similarly, the mice injected with *Rab27a* and *Pd-l1* null TRAMP-C2 cells showed a dramatically extended lifespan relative to their WT counterparts (Figure 3E). Indeed, a majority of the mice remained alive after 90 days, at which point they were used for the memory experiments described below.

To further confirm that the loss of *Rab27a* was blocking tumor growth through its role in exosome biogenesis, we repeated the experiments using *nSMase2* null cells. *nSMase2* was deleted again using CRISPR/Cas9-mediated mutagenesis (Figure S5E). Similar to PC3, *nSMase2* null TRAMP-C2 cells showed a decrease in both cellular and extracellular levels of PD-L1 (Figures S5F–S5H). These cells were injected in syngeneic mice and compared to their WT counterparts. Similar to *Rab27a* null TRAMP-C2 cells, the *nSMase2* null TRAMP-C2 cells failed to form palpable tumors during the time period when mice receiving WT cells had to be euthanized (Figure 3D). Again, a majority of the mice (8 of 10) were still alive after 90 days (Figure S3E). Together, these data show that blocking exosome biogenesis or removing PD-L1 result in a very similar tumor growth suppression phenotype.

It remained plausible that the exosome biogenesis factors were acting cell autonomously to suppress tumor growth, rather than non-autonomously via exosomal PD-L1 to suppress tumor growth via the anti-tumor immune response. To differentiate these alternative possibilities, we next asked whether the effect of *Rab27a* and/or *nSMase2* loss was dependent on an active immune system. WT, *Rab27a*, *nSMase2*, and *Pd-l1* null TRAMP-C2 cells were injected into NOD-scid IL2r_γ_^null^ (NSG)-immunodeficient mice. In striking contrast to the results seen in immunocompetent mice, the four lines led to end-stage tumors at a similar rate in the immunodeficient background (Figure 3E). Therefore, RAB27A and NSMASE2, like PD-L1, promote tumor growth through the suppression of the immune system.

### Exosomal PD-L1 Suppresses T Cell Activity in the Draining Lymph Node

**Figure 4.**
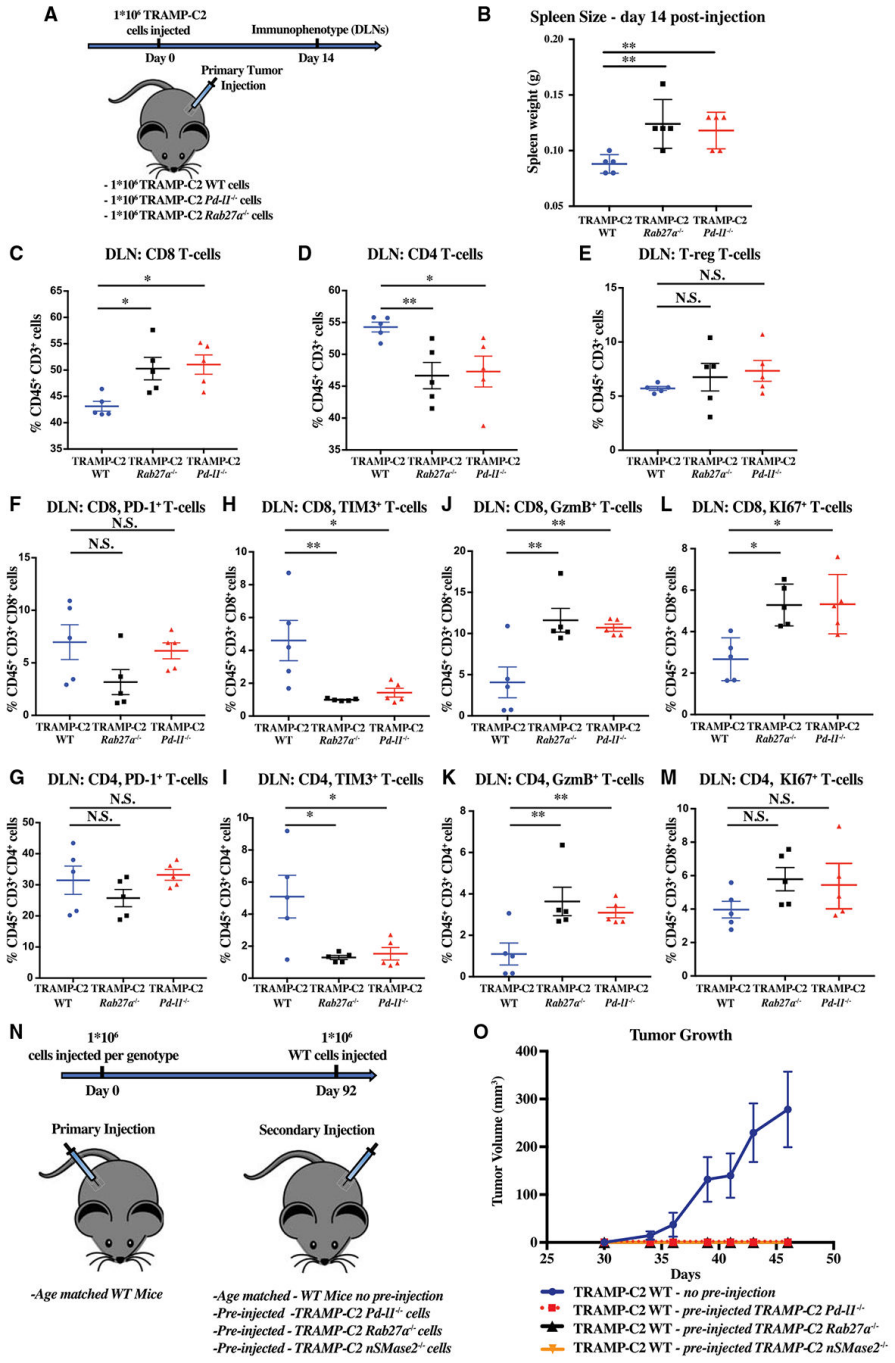
Immunophenotypes of Draining Lymph Node T Cells Predicts Immune Memory and Tumor Suppression (A) Schematic of experimental design for (B)–(M). (B) Spleen weight in grams 14 days post-injection with 1 × 10^6^ WT, *Pd-l1* null, and *Rab27a* null TRAMP-C2 cells. (C–E) Flow cytometric quantification of the percentage of CD8+ (C), CD4+ (D), and regulatory T cells (T-reg) (E), respectively, among CD45+, CD3+ cells in the draining lymph node (DLN) (n = 5 mice/genotype) 14 days post-tumor cell injection (1 × 10^6^ WT, *Pd-l1* null, and *Rab27a* null TRAMP-C2 cells). (F and G) Quantification of PD-1+ cells among CD8 (F) and CD4 (G) T cells (n = 5 mice/genotype). (H and I) Quantification of Tim3+ in CD8 (H) and CD4 (I) cells (n = 5 mice/genotype). (J–M) Quantification of granzyme B (GzmB) (J and K) or Ki67 (L and M) CD8 (J and L) and CD4 (K and M) T cells (n = 5 mice/genotype). *p < 0.5; **p < 0.01 (Student’s t test). (N) Schematic of experimental design for (O). (O) Tumor growth volume over time following secondary subcutaneous injection of 1 × 10^6^ WT TRAMP-C2 cells into immunocompetent B6 mice previously sham treated or injected with *Pd-l1*, *Rab27a*, or *nSMase2* null TRAMP-C2 cells. n = 5 for each condition. Tramp WT versus TRAMP-C2 WT pre-injected with TRAMP-C2 *Rab27a* null, p < 0.001. Tramp WT versus TRAMP-C2 WT pre-injected with TRAMP-C2 *Pd-l1* null, p < 0.001. Tramp WT versus TRAMP-C2 WT pre-injected with TRAMP-C2 *nSMase2* null, p < 0.001 (two-way ANOVA test). Error bars represent SEM. See also Figure S6.

Next, we asked whether loss of exosomes and PD-L1 have a similar effect on the immune response to the tumor. To address this question, we measured the response in the lymphoid tissues following the injection of WT, *Rab27a* null, and *Pd-l1* null TRAMP-C2 cells (Figure 4A). At 14 days, the spleens of mice injected with either mutant cell line were significantly larger than those injected with WT cells (Figure 4B). Immunophenotyping of the spleen showed equal percentages of CD8, CD4, and regulatory T cells across the three genotypes (Figure S6). These data are consistent with an enhanced generalized systemic immune response in the absence of exosomes or PD-L1. In contrast, immunophenotyping of the draining lymph nodes showed striking differences between the WT and mutant animals. CD8 positive cells made up a much greater fraction of the T cells following injection of the two mutant tumor cell lines relative to the WT line (Figure 4C). The fraction of CD4 was relatively down in the mutants, whereas FoxP3+ T regulatory cells composed a constant fraction of the T cells (Figures 4D and 4E). Given the relative increase in CD8 cells in the absence of exosomes or PD-L1, we evaluated markers of T cell exhaustion and activation within the CD8 and CD4 T cell populations. The fraction of CD8 and CD4 cells that were PD-1 high were trending down in mice receiving the mutant cells (Figures 4F and 4G). Much more significant was a decrease in the percentage of cells expressing the exhaustion marker Tim3 and increase in the percentage of cells expressing the activation marker Granzyme B (Figures 4H–4K). Furthermore, the fraction of cells positive for the proliferation marker Ki67 was significantly up among the CD8 T cells and trending up among the CD4 T cells (Figure 4L and 4M). These data are consistent with tumor-derived exosomal PD-L1 traveling to the draining lymph node and suppressing T cell activation.

We hypothesized that if exosomes are acting through PD-L1 to suppress the T cell activation in the lymph node, then in the absence of exosomal PD-L1, immune-competent mice will not only suppress immediate growth of the mutant tumor cells but will also develop memory toward the tumor cells. To test this hypothesis, mice injected with either *Rab27a* or *Pd-l1* null TRAMP-C2 cells on one flank were rechallenged 90 days later with WT TRAMP-C2 cells on the other flank (Figure 4N). Age matched mice that had not been previously injected with any tumor cells were used as control. Remarkably, WT TRAMP-C2 tumors failed to grow in any of the mice previously injected with either mutant cell line, but showed normal growth in the age matched controls (Figure 4O). Thus, exposure to tumor cells lacking exosomes or PD-L1 results in a robust memory response even against cells that secrete exosomal PD-L1. We interpret this to mean that once the T cells have been exposed to tumor antigens in the absence of exosomal PD-L1, they become resistant to the suppressive effects of exosomal PD-L1.

### Exosomal PD-L1 Promotes Growth across Different Tumor Types

**Figure 5.**
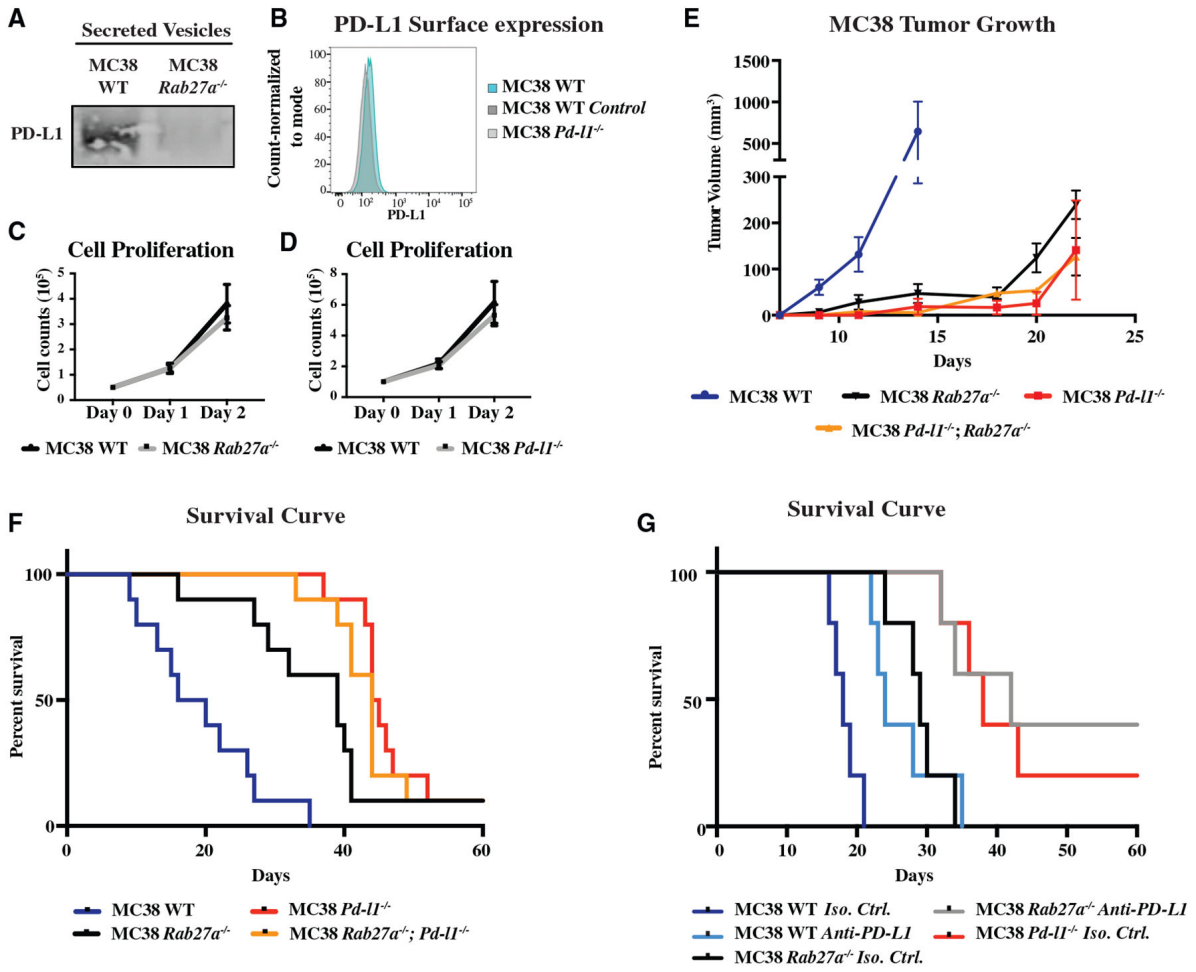
Exosomal PD-L1 Suppresses Tumor Progression in Syngeneic Colorectal Cancer Model (A) Western for PD-L1 in WT and *Rab27a* null MC38 100k g extracellular fraction. (B) Flow cytometry for surface PD-L1 on MC38 cells. (C) Cell counts over time for *Rab27a* null versus WT MC38 cells. n = 3. Error bars represent SD. (D) Cell counts over time for *Pd-l1* null versus WT MC38 cells. n = 3. Error bars represent SD. (E) Tumor growth over time following subcutaneous injection of 1 × 10^6^ WT, *Rab27a* null, or *Pd-l1* null MC38 cells into immunocompetent B6 mice. n = 5 for each genotype. Error bars represent SEM. MC38 WT versus MC38 *Rab27a* null, p < 0.05. MC38 WT versus MC38 *Pd-l1* null, p < 0.05. MC38 WT versus MC38 *Pd-l1* null*; Rab27a* null, p < 0.05 (two-way ANOVA test). (F) Mouse survival curve following injection of cells like in (D). n = 10 for each genotype. MC38 WT versus MC38 *Rab27a* null, p < 0.001. MC38 WT versus MC38 *Pd-l1* null, p < 0.001. MC38 WT versus MC38 *Pd-l1* null*; Rab27a* null, p < 0.001 (log rank test). (G) Survival curve for mice injected with WT, *Rab27a* null, or *Pd-l1* null MC38 cells followed by treatment with either anti-PD-L1 or isotype control antibody. n = 5 for each condition. MC38 WT *isotype* versus MC38 WT anti-PD-L1, p < 0.01. MC38 *Rab27a isotype* versus MC38 *Rab27a* anti-PD-L1, p < 0.05. MC38 *Pd-l1 isotype* versus MC38 *Rab27a* anti-PD-L1, ns. (log rank test). See also Figure S7.

Next, we asked whether the effect of exosomal PD-L1 on tumor progression is unique to the TRAMP-C2 model. To address this question, we evaluated a colorectal cancer model, MC38. Unlike the TRAMP-C2 model, this model shows a partial response to anti-PD-L1 and therapy (Deng et al., 2014). Western analysis showed that MC38 cells, like TRAMP-C2 cells, secrete PD-L1 (Figure 5A). They also express low levels on the cell surface (Figure 5B). Using identical guide RNAs as in the TRAMP-C2 model, *Pd-l1* and *Rab27a* were knocked out in MC38 cells via CRISPR/Cas9-mediated mutagenesis (Figures 5B and S7A). The *Rab27a* knockout cells showed a loss of PD-L1 in the secreted fraction, confirming the packaging of PD-L1 in exosomes (Figure 5A). Also, similar to TRAMP-C2 cells, the *Rab27a* and *Pd-l1* knockout MC38 cells showed no change in proliferation (Figures 5C and 5D). These cells were injected in syngeneic WT mice (Figures 5E and 5F). WT MC38 tumors grew rapidly, and mice had to be euthanized starting at 9 days. Loss of either *Rab27a* or *Pd-l1* slowed tumor growth and extended lifespan. However, unlike the TRAMP-C2 model, *Pd-l1* loss had a greater effect than *Rab27a* loss (Figure 5F). Therefore, exosomal PD-L1 appears to play an important, but partial role, in this model.

The difference in the effect of *Pd-l1* versus *Rab27a* loss allowed for an epistasis analysis to address the question of whether exosomes are acting specifically through PD-L1 to influence tumor growth. If exosomes have a PD-L1 independent role, their removal should further reduce tumor growth and extend lifespan in the *Pd-l1* null background. Therefore, we made *Rab27a; Pd-l1* double knockout MC38 cells (Figure S7). These cells grew at a rate similar to that of the *Pd-l1* single mutant cells (Figures 5E and 5F). Thus, we conclude that exosomes are functioning predominantly, if not entirely, through their presentation of PD-L1.

The difference in the effect of *Pd-l1* versus *Rab27a* loss on tumor growth also implied that there is an exosome independent pool of PD-L1 that is also functioning to suppress the immune response in this model. We hypothesized that unlike exosomal PD-L1, this pool may be sensitive to anti-PD-L1 antibody. This pool could explain why unlike the TRAMP-C2 model, the MC38 model is partially responsive to anti-PD-L1 antibodies (Deng et al., 2014). To test this hypothesis, we evaluated the effect on survival of anti-PD-L1 alone and in combination with exosome depletion. Similar to the loss of *Rab27a*, the treatment of the mice with anti-PD-L1 antibody extended survival, but not to the same degree as loss of *Pd-l1* (Figure 5G). Remarkably, the combination of *Rab27a* deletion and treatment with anti-PD-L1 antibodies lead to similar survival curve as the *Pd-l1* deletion. This finding strongly supports the conclusion that while exosomal PD-L1 is resistant to anti-PD-L1, there is another pool in this model, likely cell-surface PD-L1, which is both a significant suppressor of the anti-tumor immune response and sensitive to the antibodies.

### Exosomal PD-L1-Deficient Tumor Cells Can Suppress WT Tumor Growth at a Distance Site

**Figure 6.**
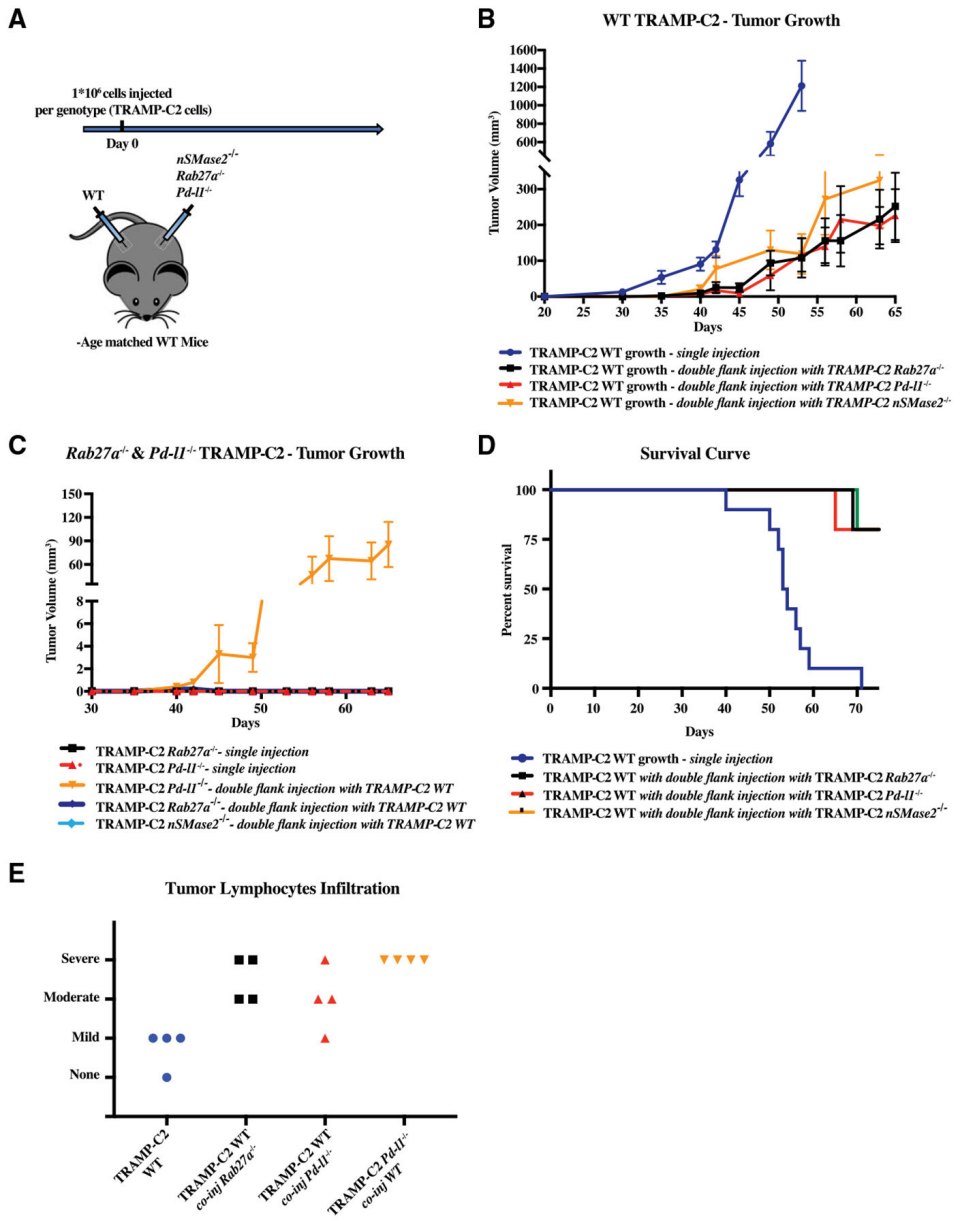
Cross-Communication between WT and Mutant TRAMP-C2 Cells at Distant Sites (A) Schematic of the experiment: briefly, immuno-competent B6 mice were co-injected with 1 × 10^6^ mutant TRAMP-C2 cells in one flank and with 1 × 10^6^ WT TRAMP-C2 cells in the other flank. (B) Tumor growth of WT TRAMP-C2 cells in mice singly injected or co-injected with either *Pd-l1* null or *Rab27a* null cells. n = 5 for each condition. Tramp WT versus TRAMP-C2 WT co-injected with TRAMP-C2 *Rab27a* null, p < 0.001. Tramp WT versus TRAMP-C2 WT co-injected with TRAMP-C2 *Pd-l1* null, p < 0.001. Tramp WT versus TRAMP-C2 WT co-injected with TRAMP-C2 *nSMase2* null, p < 0.01 (two-way ANOVA test). Error bars represent SEM. (C) Tumor growth of *Pd-l1* null or *Rab27a* null TRAMP-C2 cells in mice singly injected or co-injected with WT TRAMP-C2 cells. n = 5 for each condition. Error bars represent SEM. (D) Mouse survival curve mice singly injected with WT TRAMP-C2 cells or doubly injected with WT and *Pd-l1* null or *Rab27a* null cells. n = 5 for each genotype. (E) Histological analysis of lymphocyte infiltration of tumors under the noted conditions. Each symbol represents an individual mouse. Representative images for each state (severe, moderate, mild, and none) can be found in Figure S7E.

Exosomes can enter the blood and lymphatic systems and travel throughout the body, potentially influencing tumor growth at distant sites (Ruivo et al., 2017). Similarly, immune cells educated at one tumor site can travel throughout the body potentially influencing growth of tumors at distant sites (Chen and Mellman, 2013). Therefore, we next asked whether the simultaneous injection of WT and mutant cells at different sites would affect growth of either tumor. In particular, could exosomal PD-L1 released from WT tumor cells promote growth of mutant cells at a distant site, and/or could the T cells activated by the mutant cells suppress growth of WT cells at a distant site? To address this question, WT TRAMP-C2 cells were injected in one flank of each mouse simultaneously with *Pd-l1*, *Rab27a*, or *nSMase2* null TRAMP-C2 cells on the other flank (Figure 6A). Tumor growth on each flank was then followed over time. Remarkably, growth of the WT tumor in these double injections was dramatically reduced relative to mice where WT cells alone were injected (Figure 6B). The effects of *Pd-l1*, *Rab27a*, or *nSMase2* null cells on the distant WT tumor were similar. In contrast, the WT cells did not have any effect on the growth of *Rab27a* or *nSMase2* null cells (Figure 6C). However, the WT cells did promote the growth of *Pd-l1* null cells. This effect was small as tumor growth was much slower than the WT counterparts (Figure 6C, compare y axis to 6B). The reason for the difference between the *Pd-l1* null cells and the exosome-deficient cells is unclear and requires further study. Overall, the double-injected mice had a greatly extended survival relative to those that received WT TRAMP-C2 cells alone (Figure 6D). These findings strongly supported the notion that immune cells activated in the draining lymph node of the mutant side were able to travel and attack the WT tumor cells on the opposite flank. Indeed, histological analysis of the WT tumors showed a dramatic increase in the number of infiltrating lymphocytes when co-injected with the mutant cells on the opposite flank (Figures 6E and S7E). Together, these data show communication between the tumors, with the effect of the mutant tumor being dominant over that of the WT tumor.

### Exogenously Introduced Exosomal PD-L1 Can Rescue Immune Suppression and Tumor Growth

**Figure 7.**
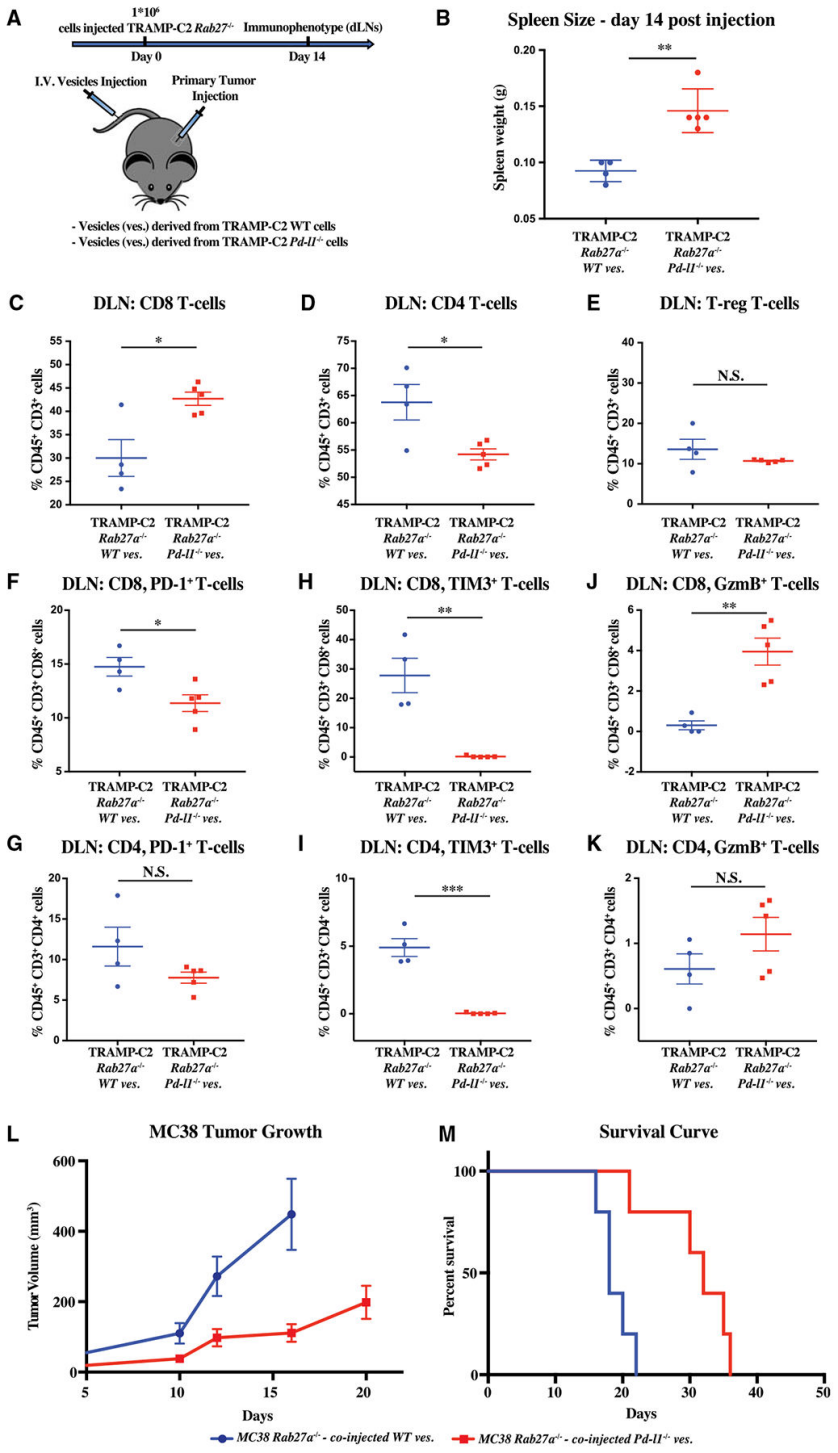
Exogenously Introduced Exosomal PD-L1 Rescues Immune Suppression and Tumor Growth of *Rab27a* Null Tumors (A) Schematic of experimental design (B)–(K); briefly, mice were transplanted with 1 × 10^6^ *Rab27a* null TRAMP-C2 cells, followed by 3 times per week tail-vein injections of exosomes that were collected from either WT or *Pd-l1* null TRAMP-C2 cells grown *in vitro*. Immune analysis was performed at 14 days. (B) Spleen weight in grams. (C–E) Flow cytometric quantification of the percentage of CD45+ CD3+ cells that are CD8 (C), CD4 (D), and regulatory T cells (T-reg) (E), respectively, in the draining lymph node (DLN). (F and G) Quantification of the percentage PD-1+ cells among the CD8 (F) and CD4 (G) T cell populations. (H and I) Quantification of the percentage of Tim3+ cells among the CD8 (H) and CD4 (I) T cell populations. (J and K) Quantification of the percentage of granzyme B (GzmB+) cells among the CD8 (J) and CD4 (K) T cell populations. (B–K) Each dot represents an individual mouse. Error bars represent mean and SD. *p < 0.05; **p < 0.01; ***p < 0.001 (Student’s t test). (L) Tumor growth over time following subcutaneous injection of 1 × 10^6^ MC38 *Rab27a* null cells followed by tail vein exosomes derived from MC38 WT and MC38 *Pd-l1* null cells grown *in vitro*. MC38 *Rab27a* null treated with WT versus *Pd-l1* null exosomes, p < 0.01 (two-way ANOVA test).(M) Survival curve following injection of cells as in (L). MC38 *Rab27a* null tumors treated with WT versus *Pd-l1* null exosomes, p < 0.01 (log rank test). (L and M) Error bars represent SEM.

Next, we asked whether exogenously introduced exosomal PDL1 could suppress the anti-tumor immune response and promote tumor growth. To address the effect on the immune response, we transplanted *Rab27a* null TRAMP-C2 cells in the flank of syngeneic mice followed by tail vein injections of *in vitro* collected exosomes from either their WT or *Pd-l1* null counterparts (Figure 7A). Exosomes from the WT, but not *Pd-l1* null, cells were able to induce a systemic immunosuppression as evidenced by nearly a 50% reduction in spleen size (Figure 7B). Furthermore, the WT exosomes were able to suppress the immune response in the draining lymph node of the *Rab27a*-deficient cells. Compared to the *Pd-l1*-deficient exosomes, the WT exosomes led to a reduced CD8/CD4 ratio, while having little effect on the T-reg cells (Figures 7C–7E). More importantly, they led to an increase in the fraction of cells expressing high levels of the exhaustion markers PD-1 and TIM3 and low levels of the activation marker Gramzyme B (Figures 7F–7K). These findings paralleled the findings seen in mice transplanted with WT versus *Rab27a* null cells (cf. Figures 4 and 7). To address the effect on the tumor growth, we transitioned to the MC38 model, given its more rapid growth characteristics. As with the TRAMP-C2 model, *Rab27a*-deficient MC38 cells were injected in the flank of syngeneic mice followed by exosome injection in the tail vein of the same mice. Consistent with the immune suppression seen in the TRAMP-C2 model, injection of exosomes collected from WT, but not *Pd-l1*-deficient MC38, cells promoted tumor growth and reduced survival (Figures 7L and 7M). These results confirm that exosomes are functioning through PD-L1 to suppress the anti-tumor immune response and thus promote tumor growth.

## DISCUSSION

All together, these data uncover a key role for exosomal PD-L1 in enabling cancer cells to evade anti-tumor immunity. Indeed, in the presence of exosomal PD-L1, T cells in the tumor’s draining lymph node express markers of exhaustion and the spleens are smaller. Genetically blocking exosome biogenesis or deleting *Pd-l1* reverses the phenotype by strongly promoting T cells activation, proliferation, and killing potential. This effect is reversed again with the introduction of exogenous exosomal PD-L1. Therefore, tumor exosomes have the ability to travel to the draining lymph node, where they present PD-L1 inhibiting T cell activation. Remarkably, blocking the release of exosomal PD-L1 not only suppresses growth of the local tumor cells but also blocks WT tumor cells injected at a distant site either simultaneously or months later. Therefore, enabling T cell activation at the local lymph node leads to a durable systemic immune response that is no longer affected by the secretion of exosomal PD-L1. The end result is extended survival of the afflicted mice.

A role for EVs, including exosomes, in tumor progression has been proposed in a number of settings (Ruivo et al., 2017). EVs can carry tumor antigens. When taken up by dendritic cells, these antigens can be presented, thereby inducing an immune response (Wolfers et al., 2001). As such, EVs were originally thought to have an anti-tumor effect. However, more recent studies have suggested a number of immunosuppressive effects. For example, EVs can inhibit dendritic cell maturation, natural killer (NK) cell function, and directly kill CD8 T cells (Abusamra et al., 2005; Liu et al., 2006; Yu et al., 2007). EVs also have been shown to promote directional migration of tumor cells (Sung et al., 2015), home tumor cells to lymph nodes (Hood et al., 2011), induce neovascularization and leakiness of tumor vessels (Peinado et al., 2012; Skog et al., 2008; Zhou et al., 2014), and, even, establish pre-metastatic niches (Fong et al., 2015; Hoshino et al., 2015). The mechanisms behind these various functions remain largely speculative.

Here, we present extensive evidence that EVs, specifically exosomes, can function to promote tumor progression by presenting PD-L1. *In vitro*, exosomes suppressed T cells in a PD-L1-dependent fashion. *In vivo*, the removal of tumor exosomes from TRAMP-C2 cells using two independent genetic mutations recapitulated the effects of deleting *Pd-l1*. These recapitulated effects included suppression of tumor growth, increased cellularity of the spleen, and the activation of a T cell response in lymph nodes with similar effects on the various activation, exhaustion, and proliferation markers. All these outcomes were reversed with the injection of *in vitro* collected exosomes carrying PD-L1. In the absence of PD-L1, the exogenously introduced exosomes had little effect. Therefore, at least in the TRAMP-C2 model, presentation of PD-L1 appears to be the major mechanism by which exosomes promote cancer progression.

This role for exosomal PD-L1 is not limited to the TRAMP-C2 model. Removal of exosomes in the colorectal MC38 model also suppressed tumor growth and extended survival. Once again, the effect was dependent on PD-L1 as the removal of exosomes had no additional effect in the *Pd-l1* null background. Interestingly though, unlike the TRAMP-C2 model, the loss of exosomes alone did not have as much of an impact as *Pd-l1* loss, suggesting a combined role of exosomal and cellular PD-L1 in the MC38 model. Remarkably, combining exosome loss with anti-PD-L1 treatment extended survival of these mice to a similar degree as removing PD-L1 altogether. These data show that in the MC38 model, both exosomal and cellular PD-L1 play an important role in promoting tumor progression in the latter, but not in the former, being sensitive to anti-PD-L1 therapy. Understanding what regulates the relative surface versus exosomal presentation of PD-L1 will be an important avenue of research going forward.

Several recent papers have identified exosomal PD-L1 in the blood of patients with a variety of cancers including head and neck cancer and melanoma (Chen et al., 2018; Theodoraki et al., 2018). In melanoma, it has been further shown that patients resistant anti-PD-1 therapy have elevated levels of exosomal PD-L1 in their blood prior to treatment (Chen et al., 2018). Therefore, our findings on the immunosuppressive and tumor-promoting roles of exosomal PD-L1 are likely to be relevant across many cancer types. However, the degree to which exosomal versus surface PD-L1 is driving immunosuppression will vary between patients and cancer types. Identifying patients who are more or less dependent on exosomal PD-L1 will be critical going forward in deciding those who are more likely to respond to therapy. Measuring levels in the blood as shown in these recent studies is likely to be one such strategy.

Going forward, targeting both cell-surface and exosome presentation of PD-L1 should be considered in any therapeutic strategy. The TRAMP-C2 model is resistant to current anti-PDL1 antibody blockade (Yu et al., 2012). However, the deletion of *Pd-l1* in the tumor cells had a striking effect. Similarly, although the MC38 model shows partial responsiveness to anti-PD-L1 therapy (Deng et al., 2014), deletion of the *Pd-l1* gene has a greater effect (Figure 5). These data are all consistent with exosomal PD-L1 being resistant to current anti-PD-L1 therapy. The reason exosomal PD-L1 is resistant is unclear. It is possible that how PD-L1 is presented on the exosome makes it less responsive to the current antibodies. Alternatively, exosomal PD-L1 may be produced at high enough levels that it can compete with the delivered antibody. It is also possible that exosomes can reach targets that are sequestered from the effects of the antibody. It will be important to tease apart the interactions of exosomal PD-L1 with current therapeutics.

Potentially the most promising therapeutic implication of our findings is that inhibition of exosome secretion at one tumor site can lead to a systemic and durable immune response against distant tumor sites or secondary tumor challenges. This observation is reminiscent of the abscopal effect, originally seen in patients treated with irradiation (Ngwa et al., 2018). In particular, irradiation of the primary tumor can lead to secondary regression of metastases. It is thought that this phenomenon is driven by activation of anti-tumor immune response and preclinical studies combining irradiation with immunosuppression are underway (Ngwa et al., 2018). It will be interesting to determine whether localized anti-exosomal therapy combined with systemic anti-PD-L1 blockade could synergize to induce a systemic immune response against multiple tumor sites simultaneously.

Here, we used genetics to dissect the role of exosomal PD-L1 in tumor progression by deleting two important exosomal biogenesis genes: *Rab27a* and *nSMNase2*. The deletion of *Rab27a* led to loss of all exosomes as measured by markers (CD63, HRS), particle tracking, and electron microscopy. The deletion of *nSMNase2* led to a loss of a majority, but not all, exosomes, as measured by the same assays. Therefore, both enzymes represent potential therapeutic targets. Small molecule inhibitors toward NSMNASE2 and RAB27A already exist, GW4869 and Nexinhib-20, respectively (Johnson et al., 2016; Luberto et al., 2002). However, these small molecules are unable to inhibit exosome release in multiple cancer cell lines, including PC3, or show an *in vivo* effect in the MC38 model (Phuyal et al., 2014) (Figures S7D–S7H), even though their respective knockouts had a profound effect (this paper). Therefore, there is a need for more effective small molecules toward these targets before any hope of developing them into drugs. Based on our findings, such drugs could have the potential to act alone or in combination with current immune-checkpoint therapies, reaching a large population of cancer patients.

In summary, we have discovered that exosomal PD-L1 is a major regulator of tumor progression through its ability to suppress T cell activation in draining lymph nodes and that its inhibition can lead to a long-lasting, systemic anti-tumor immunity.

## STAR★METHODS

### CONTACT FOR REAGENT AND RESOURCE SHARING

Further information and requests for all original resources and reagents presented in this manuscript should be directed and will be fulfilled by the Lead Contact, Robert Blelloch (robert.blelloch@UCSF.edu). MTAs may be required for some reagents per UCSF policy.

### EXPERIMENTAL MODEL AND SUBJECT DETAILS

#### Human tumor cell lines

PC3, DU145, LNCaP, SK-MEL-28 and 293T cells were obtained from ATCC, where they had been authenticated. PC3, DU145 and LNCaP are prostate cancer cell lines derived from male patients. SK-MEL-28 is a male melanoma cell line. PC3 were cultured in F-12K Medium (Kaighn’s Modification of Ham’s F-12 Medium) (GIBCO, ref 21127–022), 10% Fetal Bovine Serum (Corning, ref. 35-010-CV) and Penicillin/Streptomycin (Sigma, cat. P4333). DU145 were cultured in Eagle’s Minimum Essential Medium (EMEM) (Lonza, cat. 12–125Q), 2 mM L-glutamine (Sigma, cat. G7513), 10% Fetal Bovine Serum (Corning, ref. 35-010-CV) and Penicillin/Streptomycin (Sigma, cat. P4333). LNCaP and SK-MEL-28 were cultured in RPMI-1640 Medium (Corning, 15-040-CM), 10% Fetal Bovine Serum (Corning, ref. 35-010-CV), 2 mM L-glutamine (Sigma, cat. G7513), and Penicillin/Streptomycin (Sigma, cat. P4333). SK-MEL-28. Raji B cells and Jurkat T cells were cultured as previously reported (Hui et al., 2017). All cells were cultured at 37°C in a humidified atmosphere containing 5% CO2.

#### Mouse tumor cell lines

TRAMP-C2 cells were obtained from ATCC, where they had been authenticated, TRAMP-C2 cells were derived from a primary large T antigen expressing tumor that developed in a C57BL/6 male mouse (Foster et al., 1997). TRAMP-C2 cells were cultured in Dulbecco’s Modified Eagle’s Medium (DMEM) (UCSF cell culture facility), 5% Nu-Serum IV (Fisher, cat. CB-55004, 500 ml, to be diluted to 5%), 5% Fetal Bovine Serum (Corning, ref. 35-010-CV), Bovine Insulin (Sigma, cat. I0516), DHEA (Sigma, D-063), and Penicillin/Streptomycin (Sigma, cat. P4333). MC38 cells were kindly provided by Jeffrey Schlom’s Lab and cultured in DME H-21 (Dulbecco’s Modified Eagle Medium) High Glucose (UCSF cell culture facility), 10% Fetal Bovine Serum (Corning, ref. 35-010-CV), 1 mM sodium pyruvate (UCSF cell culture facility). MC38 cells are a colon cancer cell line derived from a female C57BL/6. All cells were cultured at 37°C in a humidified atmosphere containing 5% CO2.

#### Mice

C57BL/6J (stock # 000664) mice were purchased from “The Jackson Laboratory” and/or bred in the UCSF animal house. Immunodeficient mice NOD-Prkdc^em26Cd52^Il2rg^em26Cd22^/NjuCrl (catalog # 24110022) were obtained from Charles River. Only male mice (approximately 8–10 weeks old unless otherwise specified in text) were used for all experiments. Mice were kept under the pre-approved guidelines of the Institutional Animal Care and Use Committee at UCSF. Mice were housed in pathogen free conditions. Mice dying for nonrelated cancer causes were excluded from the studies (i.e., fights or infections). Additional information (i.e., sample size, replicates) is described in the figure legends. Each experimental group was created with mice derived from different brood.

### METHOD DETAILS

#### Cell lines treatments

To inhibit lysosome function, cells were cultured with 250 nM BafA1 (Cell Signaling, cat. 54645S) for six hours. To inhibit proteasome function, cells were cultured in 10 uM of MG132 (SIGMA, cat, M7449) for six hours.

To investigate PD-L1 glycosylation, PC3 cell lysate were treated with PNGase F (Sigma-Aldrich, cat. P7367). To investigate further glycosyl modification in the Golgi apparatus, PC3 cell lysate were treated with Endo H (NEB, cat. P0702S).

For IFN-γ treatments, 10ng/mL IFN-γ (PeproTech – human cells, cat. 300–02) (Abcam – mouse cells, cat. Ab9922) to media for 48 hours prior to exosome and cell collections.

For GW4869 treatments, 10μM GW4869 (Sigma-Aldrich, cat. D1692) to media for 48 hours prior media collections.

#### Exosome/Secreted Vesicle isolation

PC3 and SK-Mel-28 cells were plated at a density of 5 million per 15 cm plate (Corning 430599), cultured for 48 hours and media from six plates were pooled.

For TRAMP-C2 and MC38 secreted vesicle extractions, cells were plated at a density of 3 million per 15 cm plate for 48 hours and media from 6 plates were pooled.

For vesicle enrichment, media was centrifuged at 300 g for 10 minutes at room temperature, followed by 2k g for 20 min at 4°C then 12k g for 40 minutes at 4°C. Supernatant was then centrifuged twice at 100k g, for 1 hour then 10 minutes at 4°C. For exosome purification, the 100k g pellet was further spun on a sucrose gradient (20%–60% sucrose) for 16 hours at 4°C at 100k g. Fractions were collected and sucrose concentration measured using a refractometer.

It was noted that components of FBS lacked any reactivity to anti-human or anti-mouse PD-L1 and thus in experiments where only proteins were measured, vesicles were collected directly from culture media described above. In experiments where vesicles or exosomes would be counted or visualized, FBS was replaced by Knock-out Serum Replacement (KSR, GIBCO 10828028) to exclude particles present in the FBS.

#### CRISPR–Cas9-mediated gene disruption

sgRNA oligonucleotides (ELIMBIO), were cloned into pSpCas9(BB)-2A-GFP (PX458, ADDGENE) according to the published protocol (Ran et al., 2013). For each gene disrupted, two different guides were simultaneously transfected. 1ug of each vector was transfected using FUGENE HD (Promega, cat. E2311). *Pd-l1* null, *Rab27a* null and *nSMase2* null clones were obtained by GFP+ single cell cloning, 48 hours post transfection. Knockout clones were identified either by western (RAB27a) or by flow cytometry analysis for cell surface PD-L1.

**Table:** 

sgRNA oligonucleotides:	Sequences:
human *Pd-l1* guide 1:	GGTTCCCAAGGACCTATATG
human *Pd-l1* guide 2:	ACAGAGGGCCCGGCTGTTGA
human *Rab27a* guide 1:	CCAAAGCTAAAAACTTGATG
human *RAB27a* guide 2:	CAACAGTGGGCATTGATTTC
Human *nSMase2* guide 1:	GAGAAACGCAAAGGGCAGCG
Human *nSMase2* guide 2:	CGGTCCACCAGCCAGTAGCA
mouse *Pd-l1* guide 1:	GTTTACTATCACGGCTCCAA
mouse *Pd-l1* guide 2:	GGGGAGAGCCTCGCTGCCAA
mouse *RAB27a* guide 1:	CCAAGGCCAAGAACTTGATG
mouse *RAB27a* guide 2:	CACAGTGGGCATTGATTTCA
mouse *nSMase2* guide 1:	CGTTAATGGCCGACTGGCTC
mouse *nSMase2* guide 2:	AATGCCAAGTGGTTAAAGGA

#### Electron Microscopy

Exosome sucrose float fractions where prepared as described above and resuspended in PBS. Sample aliquots of 4 μL were pipetted onto 200 mesh formvar/carbon grids (EMS) which had been glow-discharged for 15 s. Samples were incubated on grids for 30 s and subsequently negatively stained with a 2% uranyl acetate solution. Data were acquired using a Philips CM200F electron microscope operating at 200 keV equipped with an UltraScan 1000 CCD Camera (Gatan).

#### Nanoparticle Tracking Analysis

200K cells were seeded per well (6 wells plate, Corning, cat. 3516) in KSR media 24 hours before collection for PC3 DU145 SK-MEL-28. 100K cells cells were seeded in KSR media 24 hours before collection for TRAMP-C2 and MC38. Media was pre-processed at 300 g for 10 minutes at room temperature, followed by 2k g for 20 min at 4°C then 12k g for 40 minutes at 4°C. The processed media was analyzed on a NanoSight LM10.

#### Antibodies

##### Immunoblotting

Anti-human PD-L1 (E1L3N) (Cell Signaling technology, cat. 13684T), anti-mouse PD-L1 (mouse) (EPR20529) (Abcam, cat. AB213480), anti-human CD63 (MEM-259) (ABCAM, cat. ab8219) GAPDH (FL-335) (Santa Cruz Biotechnology, cat. Sc25778), anti-human HRS (c-7) (Santa Cruz biotechnology, cat. SC-271455). Anti-human/mouse RAB27A (D7Z9Q) (Cell Signaling Technology, cat. 69295S). Anti-human nSMase2 (Santa Cruz Biotechnology, sc-166637)

##### Flow Cytometry

Anti-human CD274 (B7-H1) PE (MIH1) (eBioscience, cat. 125983-42), PE mouse IgG2b, k Isotype control (MG2b-57) (Biolegend, cat. 401209).

##### Immunofluorescence

Primary antibody: Anti-human PD-L1 (E1L3N) (cell signaling technology, cat. 13684T). Secondary Antibody: Anti-Rabbit Alexa Fluor 680 (Thermo Fisher Scientific, cat. A10043).

#### Western Blot Analysis

Samples were lysed in RIPA buffer (Thermo Scientific, cat. 89900) with the addition of PhosSTOP (Sigma-Aldrich, 4906837001) and Complete Mini proteasome inhibitors (Sigma-Aldrich, 05892791001). Western Blot was performed as described in the commercially available antibody protocols listed above. Western blot band intensity quantification was calculated using ImageJ.

#### Raji - Jurkat T cell stimulation assay

Raji B cells were pre-loaded by adding 30 ng/ml SEE superantigen in the media and incubating for 45 min at 37°C. Raji B cells were then washed and irradiated 10,000 rads prior to coculture. In a 96 wells plate (flat bottom) 17,000 Raji B cells were co-cultured with 70,000 Jurkat T cells. Vesicles were derived from 32 pates (15 cm plate, each plate seeded with 5 millions of cells 48 hours prior media collection) and added to the co-cultured cells as illustrated in Figure 2H. Human IL-2 was quantified from the media by ELISA (R&D Systems,#DY202–05).

#### Immunofluorescence

Cells were cultured in 8-wells chambered coverglass (Nunc, cat. 154534). Cells were fixed with 4% PFA in PBS (Fisher Scientific cat. NC9245948) and incubated for 10 minutes at room temperature. After, PBS (Sigma, cat. RNBG9030) + 0.2% of Triton-X (VWR, cat. VW8609–0) was added and the samples permeabilized in solution for 10 minutes at room temperature. Following permeabilization, samples were blocked in 5% Goat Serum (Invitrogen, cat. 16210064) PBS for 30 minutes at room temperature, and incubated at 4C overnight with primary antibodies (1:20). The following day, secondary was added (1:500) to the samples after washing, then incubated at room temperature for 3 hours. Prior imaging, sample were mounted with VECTASHIELD Antifade Mounting Medium with DAPI (Fisher Scientific, cat. NC9524612). Samples were imaged using NIKON spinning disk confocal.

#### Polysome Fractionation and RNA isolation

PC3 and SK-Mel-28 cells were pre-treated for 10 min with 100 μg/ml cycloheximide (Sigma) added in media for 10 min. Cells were washed with cold PBS (Sigma, cat. RNBG9030) with 100 μg/ml cycloheximide (Sigma, cat. C4859) and scraped. Cell pellets were lysed in 10mM Tris-HCl pH8 (Fisher Scientific, cat. BP1531), 140mM NaCl (VWR, cat. JT4058–7), 1.5mM MgCl_2_ (Thermo Fisher Scientific, cat. R0971), 0.25% NP-40 (Fisher Scientific, cat. PI85124), 0.1% Triton X-100 (VWR, cat. VW8609–0), 50mM DTT (Thermo Fisher Scientific, cat. R0861), 150 μg/ml cyclohexamide, (Sigma, cat. C4859) 640U/ml RNasin (Life Technology, cat. AM2696). After 30 min on ice, supernatant was separated by centrifugation at 9300 g and the supernatant transferred to a 10%–50% sucrose gradient. Samples were centrifuged at 35k g for 3 hours at 4C. After centrifugation the sample was fractionated (BIOCOMP). Samples were DNase treated.

#### Reverse transcriptase-quantitative PCR

DU145, PC3, LNCaP and SK-Mel-28 RNA was isolated with QIAZOL Reagent (QIAGEN, cat. 79306), and purified with Pure Link RNA mini kit (QIAGEN, 12183018A). RNA was eluted in H2O and reverse-transcribed to cDNA following the kit protocol (Thermo Scientific, cat. FERK1672). Eluted (1:50) cDNA samples were run on 7900HT fast real-time PCR system using SYBR green (Roche, cat. 04913914001).

##### Pd-l1 qPCR forward primer

GGCATTTGCTGAACGCAT, *Pd-l1* qPCR reverse primer: CAATTAGTGCAGCCAGGT (Casey et al., 2016).

##### Rpl7 qPCR forward primer

GAACCATGGAGGGTGTAGAAGAG, *Rpl7* qPCR reverse primer: GGCAAACTTCTTTCTCAGGCG.

#### Cell Fractionation

Five million PC3 cells were collected (trypsin) from a 15 cm plate. Cells were washed with 10 mL of media. Cells were then re-suspended in 5 mL of lysosome lysis buffer (1M Sucrose, 1 M Tricine pH 7.2, 0.5M EDTA pH 8, and water). Cells were spun at 900 g for 5 min at 4C. Cells were suspended up to 1.5 mL or working buffer (lysis buffer plus Protease Inhibitor, phosTOP). 1.4 ml of cell suspension were passed through cell homogenizer (Isobiotec) using a 12um ball bearing (#7,9880) for 15 times. Cell lysate were spun at 700 g for 10 minutes and the supernatant collected. Pellet was re-suspended with working buffer equivalent to the previous collected supernatant and re-spun at 700 g. Pool of supernatants was spun at 3,000 g for 15 min. Pellet was collected for western blot. The supernatant from 3kg was further spun at 25,000 g for 25 minutes in a TLA 100.3 tube (Beckman Coulter). The pellet was processed into a sucrose gradient and the supernatant was further spun at 100,000 g for 30 minutes. The pellet and the supernatant were collected for western. The pellet from the 25k g spin was spun in a sucrose gradient made of layers of 1.25M, 1.10M, and 0.25M sucrose in a TLS-55 tube (Beckman Coulter) at 120000 g for 1.5 hours. The interphase between 1.25M and 1.1M (Sucrose Light) and the pellet were collected. Both collections were run in the Western Blot.

#### Biotin-Streptavidin Assay

Cell were biotin-labeled (Sigma Aldrich, cat. B4639). Cells were washed and incubated for 24 hours in fresh media. Exosomes were then isolated from cell-conditioned media, lysed, and then biotin-labeled proteins were pulled down by streptavidin-conjugated beads (Thermo Scientific, cat. 65001).

#### Tumor cells injections

Mice were injected (S.C.) with a million TRAMP-c2 WT or TRAMP-c2 *Pd-l1* null or TRAMP-c2 *Rab27a* null or TRAMP-c2 *nSMase2* null cells. Mice were injected (S.C. with a million MC38 WT or MC38 *Pd-l1* null or MC38 *Rab27a* null cells. Mice were considered “end stage” when the tumor reached 2 cm in at least one dimension. Tumor growth was monitored three times a week by measuring tumor length and width. Tumor volume was calculated according to the following equation: length × width × 0.5 × width (Faustino-Rocha et al., 2013).

#### Mice Treatments

Where indicated, mice were treated with 200 mg antibody I.P. at days 7, 10, and 13 post tumor injection: anti–PD-L1 (10F.9G2) (BioXCell, cat. BE0101), isotype control (10F.9G2) (BioXCell, cat. LTF-2). Were indicated, mice were treated with 2.5 mg/kg GW4869 (Sigma-Aldrich, cat. D1692) from day 0 to day 14 once a day. Where indicated mice were treated with exosomes injected IV in the tail vein. 15 million cells were seeded in five 15 cm dishes (Corning CLS430599), and cultured for 48 hours. Vesicles were isolated as a 100k g pellet as described above. MC38 vesicles were resuspended in 1 mL PBS and TRAMP-C2 vesicles in 600 μl. Each mouse was injected with 100 μl of PBS containing vesicles.

#### Immune-Profiling

Mice were implanted subcutaneously (S.C.) with a million of TRAMP-C2 wt, *Pd-l1* null, or *Rab27a* null cells. Alternately, mice were injected with TRAMP-C2 *Rab27a* and treated with exosomes derived from TRAMP-C2 WT or TRAMP-C2 *Pd-l1* cells. Mice were euthanized and spleens and lymph nodes collected 14 days after tumor cell injection.

Spleens were surgically removed with sterilized surgical equipment, weighed, and crushed with the blunt end of a 10 mL syringe on Petri dishes containing 5 mL of PBS. The spleen mixtures were separately filtered through a 70 mM filter into a 50 mL conical tube, centrifuged at 1500 rpm for 5 minutes at 4°C. After wash, cell pellets were resuspended in 5mL of red blood cell lysis solution (Santa Cruz Biotechnology; Cat sc-296258) on ice for 5 minutes and stopped with the addition of 30mL of PBS. After wash, cells were reconstituted for counting by Vi-Cell (Beckman Coulter, U.S.A.). Draining lymph nodes (DLNs) were extracted with sterilized surgical equipment and crushed between the frosted surfaces of super-frosted microscope slides into wells containing PBS. Cell mixtures were then filtered through a 70 mm filter into 15 mL conical tubes. Cells were then washed and counted.

Single cell suspensions (1 million cells) were first incubated with Fc Block (Tombo Biosciences, cat. 70–0161) for 10 minutes, then co-incubated with antibodies for 20 minutes at 4°C followed by washing with staining buffer (PBS + 1% FBS). FoxP3 and intracellular staining were performed using an eBioscience intracellular kit (Cat#00-5523-00) according to the manufacturer’s protocol. Flow cytometry was performed on Fortessa X20 Dual, and data analyzed by FlowJo software (TreeStar). Details on flow cytometry antibodies used in this study can be found in Table S1.

#### Haematoxylin and eosin staining

Mice were sacrificed and the subcutaneous tumors were dissected. Tumors were fixed in 10% formalin (Fisher, cat. SF98–4) at room temperature overnight and consequentially dehydrated in 70% ethanol. Samples were mounted in paraffin blocks and sectioned at 5 μm of thickness. Haematoxylin and eosin staining was performed in Lecia Autostainer XL. Briefly, slides were baked at 60°C for 15 minutes, then treated with Xylene Substitute (Citrisolv) (Fisher chemical cat. x3p-1gal) then rehydrated and stained with Hematoxylin, followed with washes with running water. An incubation with Acid Alcohol (Sigma, cat. A3429) was performed, followed with washes with running water. The samples were then stained with eosin (Thermo Scientific) then dehydrated with ethanol followed by Xylene Substitute (Citrisolv). Samples were mounted with Permount (Fisher Scientific, cat. SP15–500). Slides were submitted for pathologic evaluation. Pathologist was blinded to sample origin (i.e., experimental versus control)

### QUANTIFICATION AND STATISTICAL ANALYSIS

Differences between groups were calculated using Student t test. Logrank test was performed for survival analysis. 2-way ANOVA was performed for tumor growth analysis. Further statistical methods are detailed in the figure legends. Data was processed with GraphPad Prism, Version 7 (GraphPad Software). In all cases a P value of 0.05 and below was considered significant: p < 0.05 (*), p < 0.01(**) and p < 0.001 (***). Details regarding number of replicates and the definition of center/error bars can be found in legends.

### DATA AVAILABILITY

Mendeley dataset: https://doi.org/10.17632/4d8h97z4c8.1

## Supplementary Material

1

2

## Figures and Tables

**Table:** KEY RESOURCES TABLE

REAGENT or RESOURCE	SOURCE	IDENTIFIER
Antibodies		
Immunoblotting: anti-human PD-L1 (E1L3N)	Cell Signaling technology	cat. 13684; RRID:AB_2687655
Immunoblotting: anti-mouse PD-L1 (mouse) (EPR20529)	Abcam	cat. AB213480
Immunoblotting: anti-human CD63 (MEM-259)	Abcam	cat. AB8219; RRID:AB_306364
Immunoblotting: anti-human/mouse GAPDH (FL-335)	Santa Cruz Biotechnology	cat. SC-25778; RRID:AB_10167668
Immunoblotting: anti-human HRS (c-7)	Santa Cruz biotechnology	cat. SC-271455; RRID:AB_10648901
Immunoblotting: Anti-human/mouse RAB27A (D7Z9Q)	Cell Signaling Technology	cat. 69295
Immunoblotting: Anti-human nSMase2 (G-6)	Santa Cruz Biotechnology	cat. SC-166637; RRID:AB_2270817
Flow Cytometry: Anti-human CD274 (B7-H1) PE (MIH1)	eBioscience	cat. 255983–42; RRID:AB_1907368
Flow Cytometry: PE mouse IgG2b, k Isotype control (MG2b-57)	Biolegend	cat. 401209
immunofluorescence: Primary antibody: Anti-human PD-L1 (E1L3N)	Cell Signaling Technology	cat. 13684; RRID:AB_2687655
immunofluorescence: Secondary Antibody: Anti-Rabbit Alexa Fluor 680	Thermo Fisher Scientific	cat. A10043; RRID:AB_2534018
Antibodies for Immunophenotyping see Table S1	This paper	N/A
anti-PD-L1 (10F.9G2)	BioXCell	cat. BE0101; RRID:AB_10949073
isotype control 971 (10F.9G2)	BioXCell	cat. LTF-2; RRID:AB_1107780
ELISA: IL-2	R&D System	cat. DY202–05
Chemicals, Peptides, and Recombinant Proteins		
BafA1	Cell Signaling	cat. 54645S
MG132	SIGMA	cat. M7449
PNGase F	Sigma-Aldrich	cat. P7367
Endo H	NEB	cat. P0702S
Human IFN-γ	PeproT ech	cat. 300–02
Mouse IFN-γ	Abcam	cat. AB9922
GW4869	Sigma-Aldrich	cat. D1692
RIPA buffer	Thermo Scientific	cat. 89900
PhosSTOP	Sigma-Aldrich	cat. 4906837001
Complete Mini proteasome inhibitors	Sigma-Aldrich	cat. 05892791001
Triton-X	VWR	cat. VW8609–0
VECTASHIELD Antifade Mounting Medium with DAPI	Fisher Scientific	cat. NC9524612
cycloheximide	Sigma	cat. C4859
Tris-HCl pH8	Fisher Scientific	cat. BP1531
NaCl	VWR	cat. JT4058–7
MgCl2	Thermo Fisher Scientific	cat. R0971
NP-40	Fisher Scientific	cat.PI85124
DTT	Thermo Fisher Scientific	cat. R0861
RNasin	Life Technology	cat. AM2696
SYBR green	Roche	cat. 04913914001
Biotin	Sigma Aldrich	cat. B4639
streptavidin-conjugated beads	Thermo Scientific	cat. 65001
Formalin	Fisher	cat. SF98–4
Xylene Substitute (Citrisolv)	Fisher chemical	cat. x3p-1gal
Acid Alcohol	Sigma	cat. A3429
Permount	Fisher Scientific	cat. SP15–500
Critical Commercial Assays		
FUGENE HD	Promega	cat. E2311
QIAZOL Reagent	QIAGEN	cat. 79306
Pure Link RNA mini kit	QIAGEN	cat. 12183018A
Thermo Scientific Maxima First Strand cDNA Synthesis Kit for RT-qPCR	Thermo Scientific	cat. FERK1672
Red blood cell lysis solution	Santa Cruz Biotechnology	cat. SC-296258
Zombie UV Fixable Viability Kit	Biolegend	cat. 423107
Intracellular kit	eBioscience	cat. 00-5523-100100
Deposited Data		
Uncropped Western Blots	Mendeley dataset	https://doi.org/10.17632/4d8h97z4c8.1
Experimental Models: Cell Lines		
PC3	ATCC	CRL-1435; RRID:CVCL_0035
DU145	ATCC	HTB-81; RRID:CVCL_0105
LnCap	ATCC	CRL-1740; RRID:CVCL_1379
SK-MEL-28	Robert Judson Lab (UCSF)	RRID:CVCL_0526
293 T	ATCC	CRL-3216; RRID:CVCL_0063
RAJI B CELLS	Ron Vale Lab (UCSF)	RRID:CVCL_0511
JURKAT T-CELLS Overexpressing PD-1	Ron Vale Lab (UCSF)	https://doi.org/10.1126/science.aaf1292
RAJI B CELLS Overexpressing PD-L1	Ron Vale Lab (UCSF)	https://doi.org/10.1126/science.aaf1292
TRAMP-C2	ATCC	CRL-2731; RRID:CVCL_3615
MC38	Jeffrey Schlom lab (NCI)	RRID:CVCL_B288
Experimental Models: Organisms/Strains		
C57BL/6J	The Jackson Laboratory	RRID:IMSR_JAX:000664
NCG (NOD-Prkdc^em26Cd52^Il2rg^em26Cd22^/NjuCrl)	Charles River Laboratories	RRID:IMSR_CRL:572
Oligonucleotides		
Sequences for sgRNAs, see table in STAR Methods	This paper	N/A
*Pd-l1* qPCR forward primer: GGCATTTGCTGAACGCAT	Casey et al., 2016	https://doi.org/10.1126/science.aac9935
*Pd-l1* qPCR reverse primer: CAATTAGTGCAGCCAGGT	Casey et al., 2016	https://doi.org/10.1126/science.aac9935
Rpl7 qPCR forward primer: GAACCATGGAGGGTGTAGAAGAG	This paper	N/A
Rpl7 qPCR reverse primer: GGCAAACTTC lllCTCAGGCG	This paper	N/A
Recombinant DNA		
pSpCas9(BB)-2A-GFP	ADDGENE	PX458
pMH243-CD63-GFP	Hebrok Lab (UCSF)	N/A
Software		
FlowJo	10.5.2	https://www.flowjo.com/
Prism	7.0b	https://www.graphpad.com/scientific-software/prism/
ImageJ	1.49V	https://imagej.nih.gov/ij

## Highlights

- Genetic blockade of exosomal PD-L1 extends survival by promoting anti-tumor immunity
- Exosomal PD-L1 suppresses T cell activity in the draining lymph node
- Exosome-deficient tumor cells induce systemic anti-tumor immunity and memory
- Exosomal PD-L1 appears to be resistant to anti-PD-L1 antibody blockade
