# Supplementary material for: Suppression of Exosomal PD-L1 Induces Systemic Anti-tumor Immunity and Memory
Source: Cell. Author manuscript; Available in PMC 2019 May 3. (PMC6499401; doi:10.1016/j.cell.2019.02.016)
Supplement: 1 [file NIHMS1526616-supplement-1.pdf]

Cell, Volume *177*

Supplemental Information

Suppression of Exosomal PD-L1 Induces Systemic Anti-tumor Immunity and Memory

Mauro Poggio, Tianyi Hu, Chien-Chun Pai, Brandon Chu, Cassandra D. Belair, Anthony Chang, Elizabeth Montabana, Ursula E. Lang, Qi Fu, Lawrence Fong, and Robert Blelloch

Supplemental Table 1: T cell Panel (Related to STAR Methods)					
**Surface Staining**					
**Marker**	**Laser**	**Color**	**Channel**	**Company**	**Cat. No.**
CD4	**Violet C**	**BV711**	V710/50	Biolegend	100447 RRID:AB_2564586
CD8	**Violet E**	**BV605**	V610/20	Biolegend	100744 RRID:AB_2562609
CD45	**Violet G**	**BV510**	V515/20	Biolegend	103138 RRID:AB_2563061
*Tim-3	**Blue B**	**PerCp-Cy5.5**	B710/50	Biolegend	119718 RRID:AB_2571935
CD3e	**UV G**	**BUV395**	U379/28	BD Biosciences	563565
PD-1	**Violet D**	**BV650**	V450/50	BD Biosciences	135215 RRID:AB_10696422
Live/Dead	**UV E**	**Live/dead**	U515/30	Biolegend	423107
Fc				Tonbo Bioseciences	70-0161
**Intracellular Staining**					
Ki67	**Violet H**	**BV480**	V470/15	BD Biosciences	566109
GzB	**Red B**	**AF700**	R730/45	BD Biosciences	560213 RRID:AB_1645453
Foxp3	**Blue D**	**FITC**	B515/20	eBiosciences	11-5773-82 RRID:AB_465243
**Surface Staining FMO**					
**Marker**	**Laser**	**Color**	**Channel**	**Company**	**Cat. No.**
CD4	**Violet C**	**BV711**	V710/50	Biolegend	100447 RRID:AB_2564586
CD8	**Violet E**	**BV605**	V610/20	Biolegend	100744 RRID:AB_2562609
CD45	**Violet G**	**BV510**	V515/20	Biolegend	103138 RRID:AB_2563061

*Tim-3	**Blue B**	**PerCp-Cy5.5**	B710/50	Biolegend	119718 RRID:AB_2571935
CD3e	**UV G**	**BUV395**	U379/28	BD Biosciences	563565
PD-1	**Violet D**	**BV650**	V450/50	BD Biosciences	135215 RRID:AB_10696422
Live/Dead	**UV E**	**Live/dead**	U515/30	Biolegend	423107
Fc				Tonbo Biosceciences	70-0161
**Intracellular Staining FMO**					
Ki67	**Violet D**	**BV650**	V660/20	BD Biosciences	566109
GzB	**Red B**	**AF700**	R730/45	BD Biosciences	560213 RRID:AB_1645453
Foxp3	**Blue D**	**FITC**	B515/20	eBiosciences	11-5773-82 RRID:AB_465243
