# Supplementary material for: Suppression of Exosomal PD-L1 Induces Systemic Anti-tumor Immunity and Memory
Source: Cell. Author manuscript; Available in PMC 2019 May 3. (PMC6499401; doi:10.1016/j.cell.2019.02.016)
Supplement: 2 [file NIHMS1526616-supplement-2.pdf]

Supplemental Figures

Cell

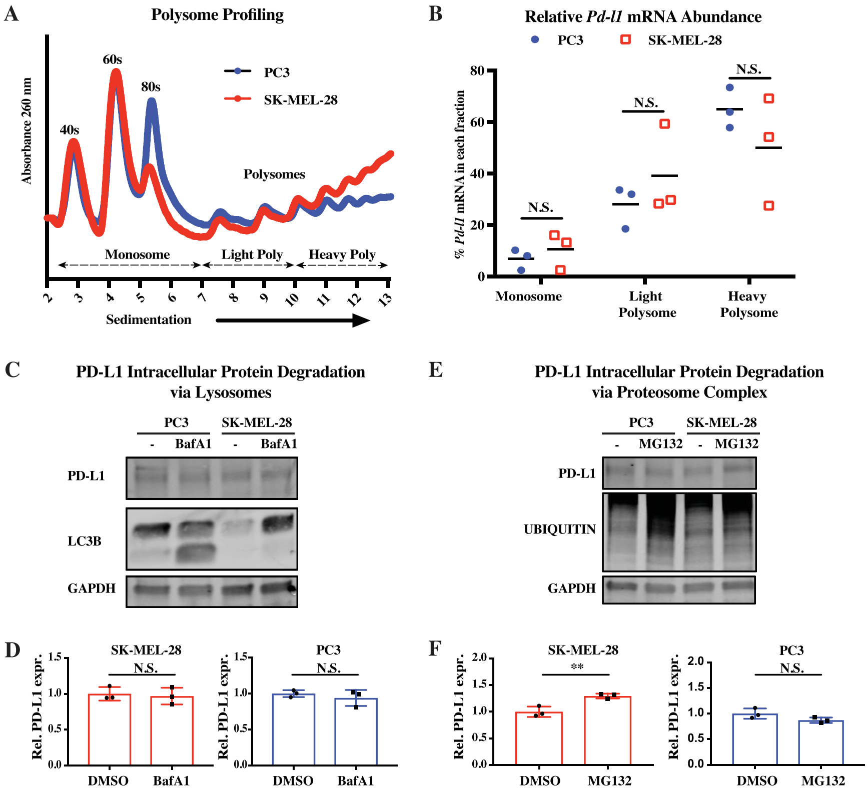

Figure S1. Differences in Translation Rates nor Protein Degradation Can Explain Discordance of mRNA and Protein Levels between PC3 and SK-MEL-28 Cells, Related to Figure 1

(A) Polysome profiles in PC3 and SK-MEL-28 cells.

(B) Distribution of Pd-l1 mRNA across the monosome fractions (2-6), low polysome fractions (7-9), and heavy polysome fractions (8-12) in PC3 and SK-MEL-28 cells.

(C) Representative western for PD-L1 in PC3 and SK-MEL-28 cells treated with or without the lysosome inhibitor Bafilomycin A1 (BafA). LC3B, positive control. GAPDH, loading control.

(D) Quantification of three independent experiments in each cell line.

(E) Representative western for PD-L1 in PC3 and SK-MEL-28 cells treated with or without the proteasome inhibitor MG132. Ubiquitin, positive control. GAPDH, loading control.

(F) Quantification of three independent experiments in each cell line. **$p < 0.01$. N.S., not significant.

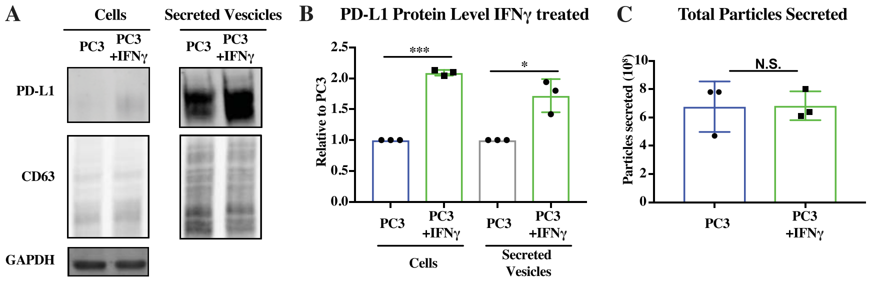

Figure S2. IFN-γ Increases PD-L1 Levels in Both Cells and Extracellular Vesicles, without Affecting Number of Vesicles or Exosomes, Related to Figure 1

(A) Representative western for PD-L1 in PC3 cells and 100k g pellet, plus and minus IFN-γ. CD63 and GAPDH, loading controls.

(B) Quantification of PD-L1 in westerns. PD-L1 is normalized to GAPDH for cells and to cell number for exosomes. n = 3.

(C) NanoSight measurement of total number of vesicles secreted plus and minus IFN-γ. n = 3. Error bars represent SD. *$p < 0.05$, ***$p < 0.001$. N.S., not significant.

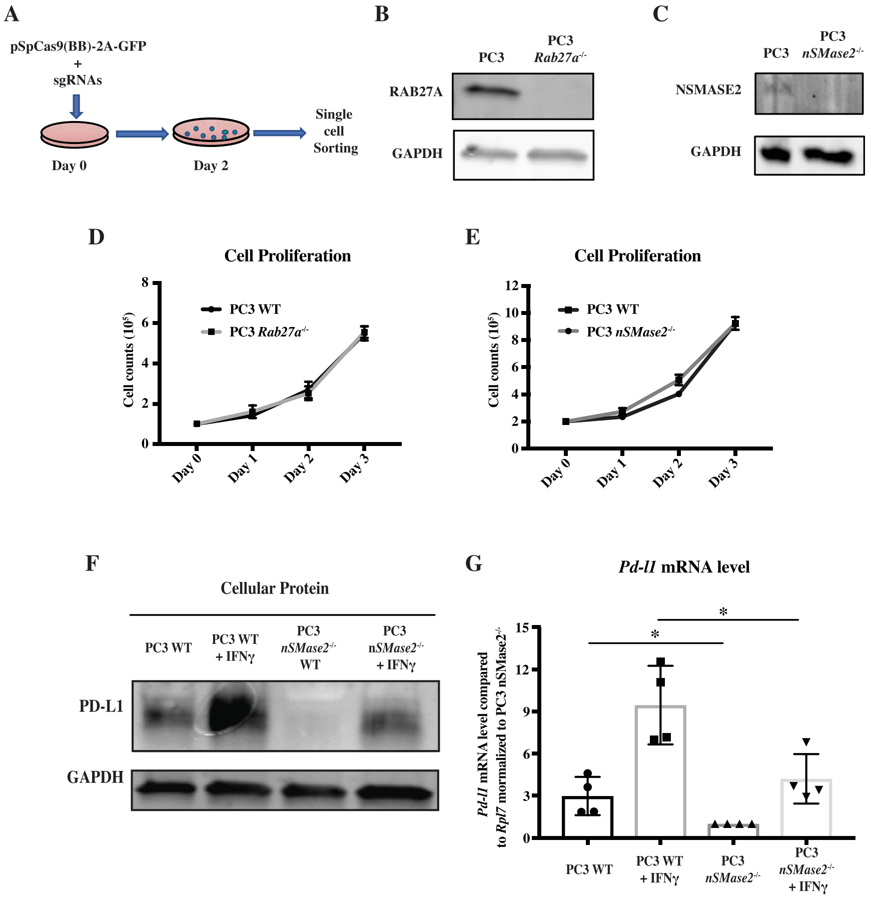

Figure S3. Deletion of *Rab27a* and *nSMase2* in PC3 Cells, Related to Figure 2

(A) Schematic for CRISPR/Cas9-mediated deletion of *Rab27a* and *nSMase2*.
(B) Western for RAB27A in WT and *Rab27a* null PC3 cells.
(C) Western for NSMASE2 in WT and *nSMase2* null PC3 cells.
(D) Cell counts of WT versus *Rab27a* null PC3 cells over period of 3 days.
(E) Cell counts of WT versus *nSMase2* null PC3 cells over period of 3 days.
(F) Western for cellular PD-L1 in WT and *nSMase2* null PC3 cells treated or untreated with INF-γ.
(G) qPCR for Pd-l1 mRNA level relative to RPL7 in WT and *nSMase2* null PC3 cells n = 4. *$p < 0.05$, error bars represent SD.

Cell

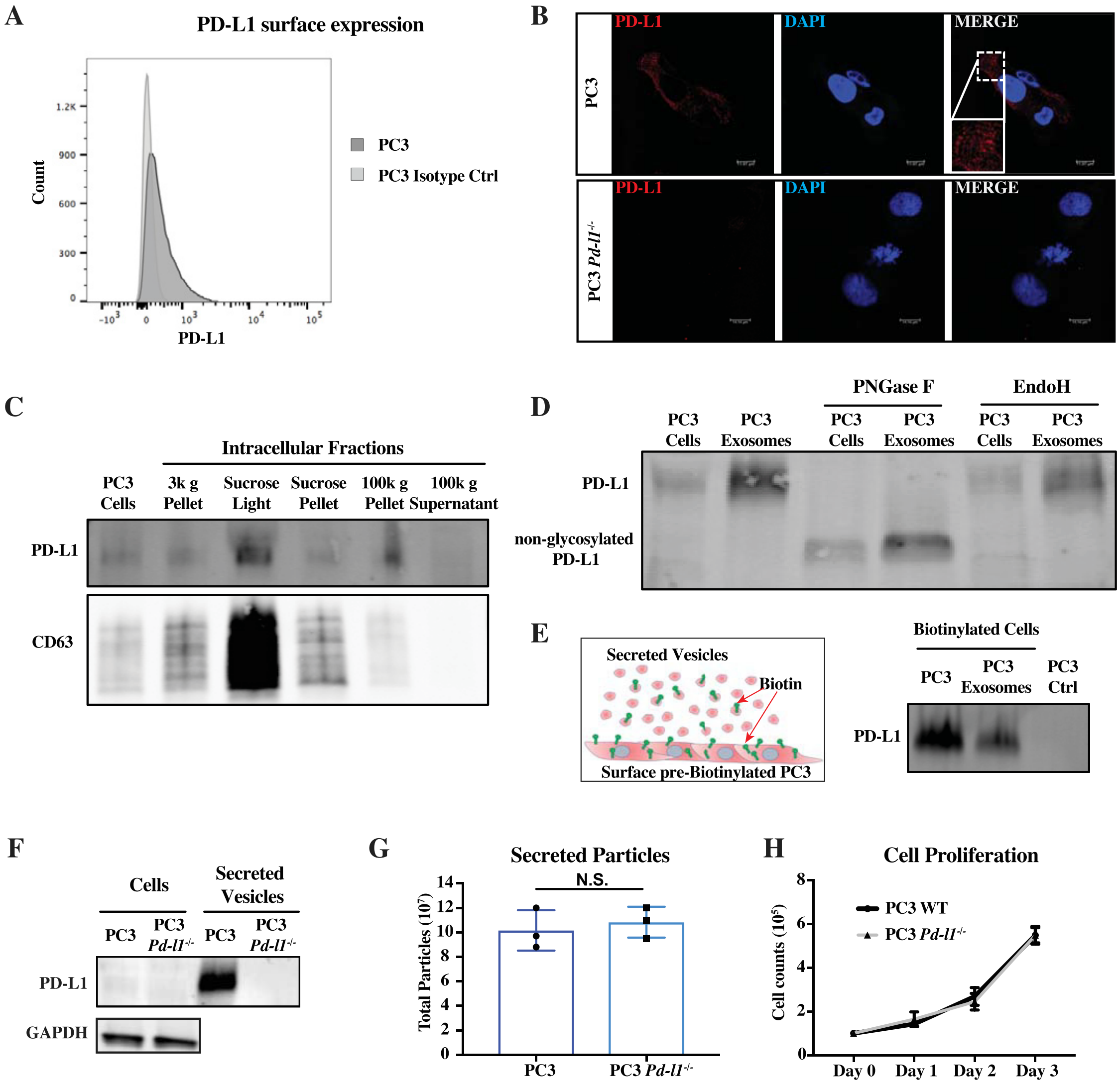

Figure S4. Exosomal PD-L1 Arises from Endocytosis of Cell Surface PD-L1, Related to Figure 2

(A) Flow cytometry for surface PD-L1 on PC3 cells.

(B) Confocal images of WT and *Pd-l1* null PC3 cells stained with anti-PD-L1 and Dapi, showing cytoplasmic PD-L1 punctae consistent with vesicular staining.

(C) Western for PD-L1 and CD63 following cellular fractionation based on size and density. PD-L1 is enriched in endolysomal fraction, marked by high levels of CD63.

(D) Western following treatment with different endoglycosidases. PNGaseF removes all N-linked glycosylation. In contrast, EndoH is blocked by side chain modifications occurring in the Golgi.

(E) Cell surface biotinylatin assay. Left: schematic of assay. Right: Western for PD-L1 following strepavidin pull down from cells and exosomes, 48 hours following cell surface labeling. Control, not biotinylated PC3 cells.

(F) Western for PD-L1 in *Pd-l1* null PC3 cells and 100k g extracellular fraction from those cells.

(G) Nanoparticle quantification of vesicles from WT and *Pd-l1* null PC3 cells. n = 3.

(H) Cell counts over time for WT and *Pd-l1* null PC3 cells. n = 3. Error bars represent SD. $^{*}p < 0.05$, $^{**}p < 0.01$, $^{***}p < 0.001$.

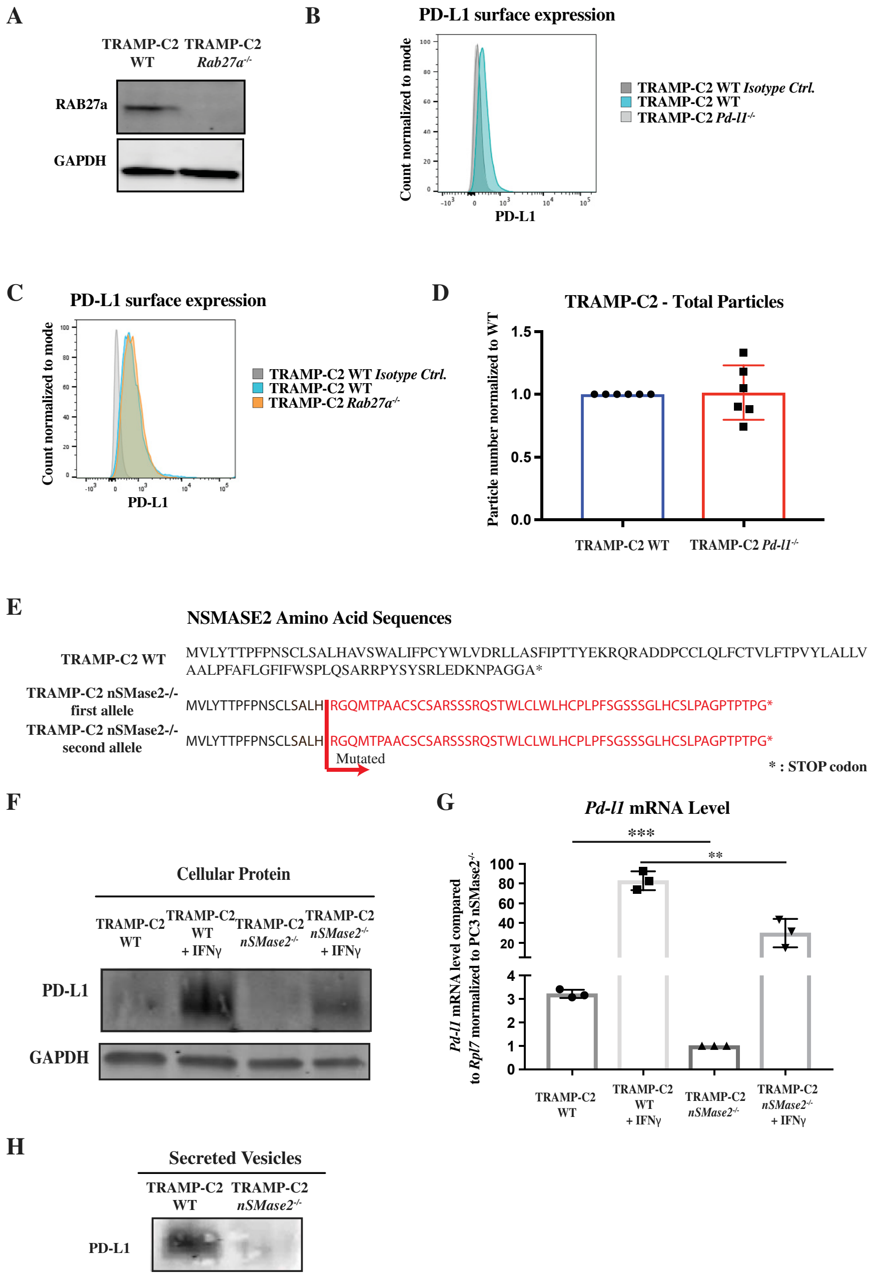

Figure S5. Characterization of *Rab27a* and *nSMase2* Knockout TRAMP-C2 Cells, Related to Figure 3

(A) Western for RAB27A in WT and *Rab27a* null TRAMP-C2 cells.

(B and C) Flow cytometry for cell surface PD-L1 in WT, *Pd-l1*, and *Rab27a* null TRAMP-C2 cells.

(D) Particles secreted by TRAMP-C2 WT and TRAMP-C2 *Pd-l1* cells.

(E) CRISPR/Cas9 mediated mutations induce frameshift and early stop in *nSMase2* gene.

(F) Western for cellular PD-L1 in WT and *nSMase2* null TRAMP-C2 cells treated or untreated with INF-γ.

(G) qPCR for *Pd-l1* mRNA level relative to *Rpl7* in WT and *nSMase2* null TRAMP-C2 cells. n = 3. Error bars = SD.

(H) Western for secreted PD-L1 in WT and *nSMase2* null TRAMP-C2 cells.

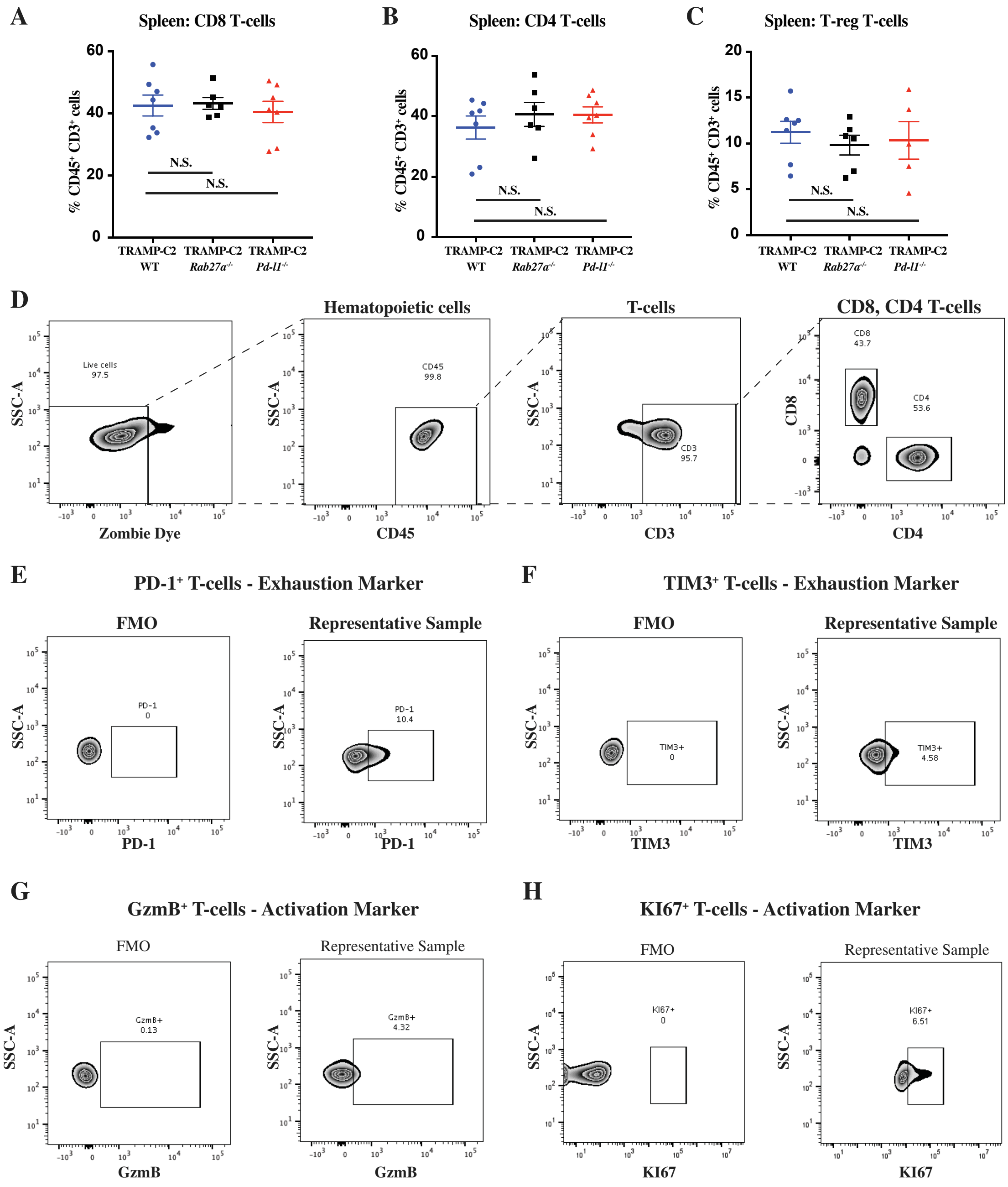

Figure S6. Immune Profiling of Spleen and Gating Strategy, Related to Figure 4

(A–C) Flow analysis of indicated subsets of CD8+ (A), CD4+ (B) and regulatory (T-reg) (C) T cells in spleen among CD45+ CD3+ cells (n = 5 mice/genotype) 14 days post-tumor injection ($1*10^6$ WT, *Pd-l1* null and *Rab27a* null TRAMP-C2 cells). Error bars = SEM. N.S., not significant.

(D) Gating strategies for flow analysis of CD45+ CD3+ T cells.

(E–H) Representative flow plots of T cells stained for PD-1 (E) Tim3 (F), GzmB (G) and Ki67 (H) over FMO.

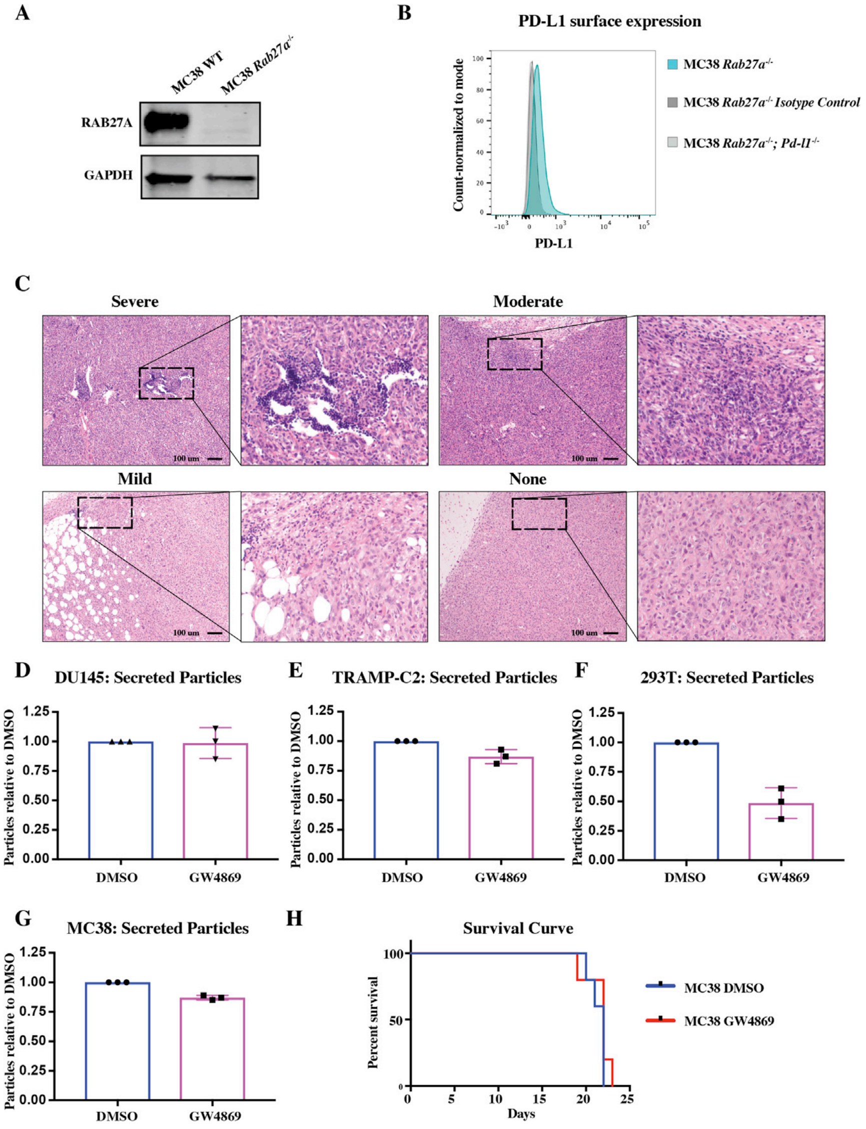

Figure S7. Characterization of *Pd-l1* and *Rab27a* Knockout MC38 Cells, Representative H&E of Lymphocyte Infiltration, and GW4869 Treatments, Related to Figures 5 and 6

(A) Western for RAB27a in WT and *Rab27a* knockout MC38 cells.

(B) Flow cytometry for cell surface PDL1 in *Rab27a* knockout and *Rab27a ;Pd-l1* double knockout MC38 cells. For double knockouts, *Pd-l1* was mutated in a background of *Rab27a* null cells.

(C) Representative H&E for each category of lymphocyte infiltration show in Figure 6E.

(D–G) NanoSight measurement of vesicle number secreted with or without GW4869 treatment in DU145 (D), TRAMP-C2 (E), 293T (F) and MC38 (G) cell lines.

(H) Mouse survival curve following MC38 tumor treated with GW4869 or DMSO as control. n = 5 mice for each group.
